# Promoter Activation in Δ*hfq* Mutants as an Efficient Tool for Specialized Metabolite Production Enabling Direct Bioactivity Testing

**DOI:** 10.1002/anie.201910563

**Published:** 2019-12-12

**Authors:** Edna Bode, Antje K. Heinrich, Merle Hirschmann, Desalegne Abebew, Yan‐Ni Shi, Tien Duy Vo, Frank Wesche, Yi‐Ming Shi, Peter Grün, Svenja Simonyi, Nadine Keller, Yvonne Engel, Sebastian Wenski, Reuel Bennet, Sophie Beyer, Iris Bischoff, Anthony Buaya, Sophie Brandt, Ibrahim Cakmak, Harun Çimen, Simone Eckstein, Denia Frank, Robert Fürst, Martin Gand, Gerd Geisslinger, Selcuk Hazir, Marina Henke, Ralf Heermann, Virginie Lecaudey, Wilhelm Schäfer, Susanne Schiffmann, Anja Schüffler, Rebecca Schwenk, Marisa Skaljac, Eckhard Thines, Marco Thines, Thomas Ulshöfer, Andreas Vilcinskas, Thomas A. Wichelhaus, Helge B. Bode

**Affiliations:** ^1^ Mol. Biotechnol. Univ. Frankfurt 60438 Frankfurt Germany; ^2^ LOEWE-TBG 60325 Frankfurt Germany; ^3^ Senckenberg BiK-F 60325 Frankfurt Germany; ^4^ Cell Biology and Neuroscience Department of Developmental Biology of Vertebrates 60438 Frankfurt Germany; ^5^ Pharmaceutical Biology Faculty of Biochemistry, Chemistry and Pharmacy University of Frankfurt 60438 Frankfurt Germany; ^6^ Mol. Phytopathol. Univ. Hamburg 22609 Hamburg Germany; ^7^ Aydin Adnan Menderes University Department of Plant Protection 09100 Aydin Turkey; ^8^ Aydin Adnan Menderes University Department of Biology 09100 Aydin Turkey; ^9^ Universität Mainz Molekulare Physiologie, Mikrobiologie und Weinforschung 55128 Mainz Germany; ^10^ University Hospital Frankfurt Medical Microbiology and Infection Control 60596 Frankfurt Germany; ^11^ Current address: Food Chemistry and Food Biotechnology University of Giessen 35392 Giessen Germany; ^12^ Fraunhofer Inst. for Mol. Biol. and Appl. Ecology Branch for Translat. Med. and Pharmacology 60596 Frankfurt Germany; ^13^ Univ. Hospital Clin. Pharmacol. 60590 Frankfurt Germany; ^14^ IBWF gGmbH Biotechnology and Drug Research 67663 Kaiserslautern Germany; ^15^ Fraunhofer Institute for Molecular Biology and Applied Ecology Branch for Bioresources 35394 Giessen Germany; ^16^ Microbiology and Wine Research at the Institute of Molecular Physiology (IMP) University of Mainz 55128 Mainz Germany; ^17^ Univ. Frankfurt Ecology, Evolution and Diversity 60438 Frankfurt Germany; ^18^ Univ. Giessen Insect Biotechnology 35392 Giessen Germany; ^19^ BMLS Univ. Frankfurt 60438 Frankfurt Germany

**Keywords:** bioactivity testing, easyPACId, natural products, proteobacteria, simplified production

## Abstract

Natural products (NPs) from microorganisms have been important sources for discovering new therapeutic and chemical entities. While their corresponding biosynthetic gene clusters (BGCs) can be easily identified by gene‐sequence‐similarity‐based bioinformatics strategies, the actual access to these NPs for structure elucidation and bioactivity testing remains difficult. Deletion of the gene encoding the RNA chaperone, Hfq, results in strains losing the production of most NPs. By exchanging the native promoter of a desired BGC against an inducible promoter in Δ*hfq* mutants, almost exclusive production of the corresponding NP from the targeted BGC in *Photorhabdus*, *Xenorhabdus* and *Pseudomonas* was observed including the production of several new NPs derived from previously uncharacterized non‐ribosomal peptide synthetases (NRPS). This *easy*PACId approach (*easy* Promoter Activated Compound Identification) facilitates NP identification due to low interference from other NPs. Moreover, it allows direct bioactivity testing of supernatants containing secreted NPs, without laborious purification.

## Graphical abstract

**From all to nothing and back to just one**! Promoter exchange in Δ*hfq* mutants of proteobacteria allows the generation of strains that produce exclusively only one natural product (NP) class facilitating NP isolation and structure elucidation and even direct bioactivity testing of crude extracts.

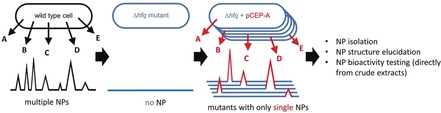

## Introduction

Natural products (NPs), also known as secondary or specialized metabolites, are produced by almost all bacteria, archaea and fungi. They fulfill numerous functions as part of their ecology acting for example as antibiotics, siderophores, toxins or signals mediating all aspects of organismic interaction between the microbes and their environment.^1^, ^2^ NPs and chemical derivatives thereof are also central to our health and agriculture, being applied as clinically‐relevant antibiotics, immunosuppressants, anticancer, antiviral drugs or as pesticides.^3^ Their biological properties are a result of their chemical structures that have been optimized during evolution towards a specific target. Hence, they represent a rich source of promising leads for new drugs capable of overcoming microbial resistances and to fight emerging diseases.

The ever‐increasing number of sequenced microbial genomes has created a number of resources and repositories for mining the data, with a particular emphasis on the identification of biosynthetic gene clusters (BGCs) involved in NP production.^4^, ^5^ In most cases, the number of these BGCs encoded in the genomes far outnumbers the quantity of NPs produced under laboratory conditions. How to exploit the potential of this hidden chemical diversity and consequently deliver pure NPs in a simple, rapid and cost‐efficient method, gaining sufficient amounts of NPs for broad bioactivity testing is a major scientific challenge.

Different strategies have been implemented for the activation of these BGCs that sometimes might not be expressed under laboratory conditions and therefore are considered “silent”. Methods for BGC activation range from varying cultivation conditions (also called the OSMAC approach) to co‐cultivation approaches.^2^, ^6^, ^7^ An individual BGC can also be activated using deletion/overexpression of global (or specific) transcription factors,^7^, ^8^ application of transcription factor decoys^9^ or promoter exchange approaches activating these BGCs using inducible promoters.^10^, ^11^ Heterologous expression of a complete BGC has also been applied successfully for NP production.^12^, ^13^, ^14^ However, many challenges remain with all of these methods and particularly with heterologous hosts that may lack required building blocks (e.g. fatty acids, amino acids) for proper biosynthesis of the original NP. Furthermore, expression levels may be low due to toxicity against the heterologous producer.^15^

**Figure 1:**
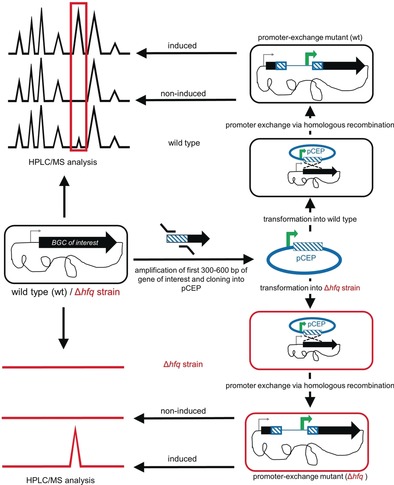
Schematic overview showing the outcome of promoter exchange for a desired biosynthetic gene cluster (BGC) in wild type (top) and Δ*hfq* mutants (bottom) using integrative pCEP plasmids.^10^

The drawback of all described approaches is that the NP of interest is generated in addition to undesired NPs that are also produced under any given condition. The resulting complex NP mixture might be very difficult to separate. Ideally, the activation of a single BGC would result in the production of a single corresponding NP and its derivatives. In the prolific NP producing bacterial genus *Photorhabdus*, we recently showed a dependence of NP production on the RNA chaperone, Hfq, that modulates BGC expression through sRNA/mRNA interactions.^16^ In a Δ*hfq* strain, the biosynthesis of NPs is almost completely lost. Here we show that activation of desired BGCs in a Δ*hfq* background led to the nearly exclusive production of the corresponding NPs in several proteobacteria following targeted BGC activation using the inducible promoter P_*BAD*_. Compared to BGC activation in wild type strains, or approach termed *easy*PACId (*easy* Promoter Activated Compound Identification) leads to culture supernatants lacking most undesired NPs, thereby enabling not only simplified identification and purification of the desired NP, but also direct bioactivity testing of culture extracts or supernatants against different target organisms, without time‐consuming NP purification (Figure 1).

## Results and Discussion

**Figure 2:**
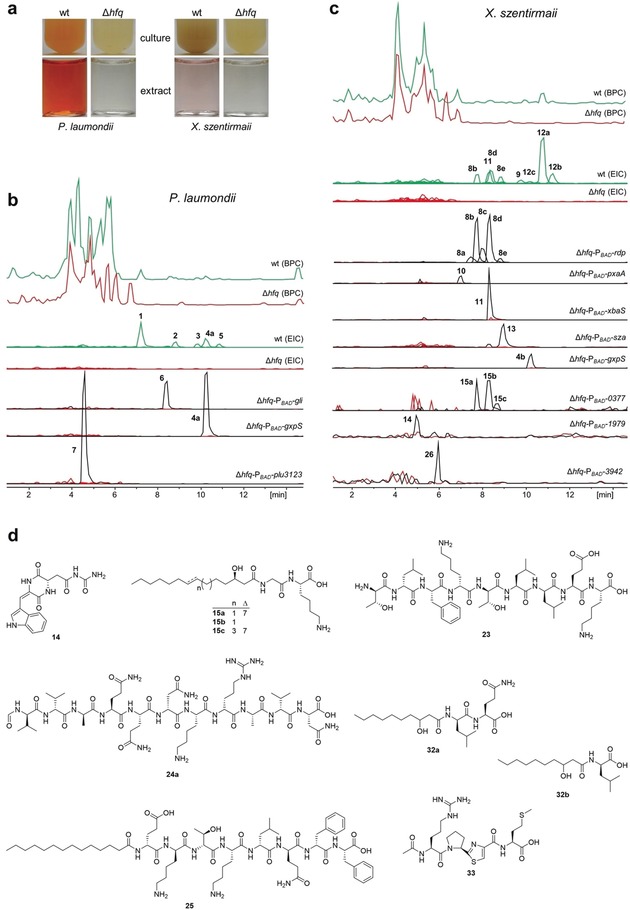
Promoter exchange in Δ*hfq* results in specific NP production. a) Culture supernatants (top) and XAD‐16 extracts (bottom) of wild type (wt) and Δ*hfq* mutants of *P. laumondii* TTO1 and *X. szentirmaii*. HPLC/MS analysis of *P. laumondii* TTO1 (b) and *X. szentirmaii* (c) wt and Δ*hfq* mutants are shown as base peak chromatograms (BPC). For better visualization extracted ion chromatograms (EICs) representing major derivatives of all known NP classes in both strains (Supplementary Table 1, Supplementary Figure 1) are shown at the bottom. For **14** and **26**, the production titer was still very low compared to other NPs that only EICs of induced (red) and non‐induced Δ*hfq* mutants (black) are shown. Both compounds were not detected in the wt. d) Structures of identified new NPs from *X. szentirmaii* (**14**, **15**), *Photorhabdus* PB45.5 (**23**, **24 a**), *Xenorhabdus* KJ12.1 (**25**) and *Pseudomonas entomophila* L48 (**32**, **33**). The chiral centers of hydroxyl groups and amino acid residues in new NPs were predicted by analyzing the corresponding BGCs (for details see Supplementary Information A.5 and Supplementary Figure 3).

Compared to the wild type *Photorhabdus laumondii* TTO1 and *Xenorhabdus szentirmaii,* Δ*hfq* mutants appear colorless (Figure 2 a) due to the absence of their main pigments, anthraquinones (**1**) and phenazines, respectively (see Supplementary Figure 1 and Supplementary Table 1 for all NP structures). HPLC‐MS analysis of culture supernatants confirmed the absence of all NPs in the Δ*hfq* strains compared to WT when extracted ion chromatograms (EICs) of NPs were analyzed (Figure 2 b,c). Although in some Proteobacteria like *Pseudomonas aeruginosa* deletion of *hfq* results in a growth defect compared to the respective wild type,^17^ this was hardly observed for *Photorhabdus* and *Xenorhabdus* (Supplementary Figure 2).

### Targeted NP production in Xenorhabdus and Photorhabdus Δhfq mutants

In *P. laumondii* TTO1‐Δ*hfq,* the known NPs^18^, ^19^ GameXPeptide A (**4 a**), glidobactin A (**6**), and ririwpeptide A (**7**) and in *X. szentirmaii*‐Δ*hfq* GameXPeptides (**4**), rhabdopeptides (**8**) and pyrrolizixenamides (**10**) were individually produced upon promoter activation following genomic integration of the non‐replicating pCEP plasmids carrying the first 600 bp of the first gene in the BGC of interest behind the inducible P_*BAD*_ promoter (Figure 1). For NPs that are also produced in wild type strains (e.g. **4**, **8**) their activation in a Δ*hfq*‐mutant often leads to a strong increase in the production titer^10^ but with very little undesired NPs from other BGCs being produced. Furthermore, it allows the activation of BGCs that seem silent under the cultivation conditions used (**6**, **7**, **10**, **14**, **15 a**–**c**, **27**). The independence of the induced promoter from (often unknown) intracellular regulation mechanisms may be a reason for this overproduction as well as the increased availability of building blocks due to all other NP pathways being inactive.

Promoter exchange of Xsze_03460 and Xsze_03680 in *X. szentirmaii*‐Δ*hfq* led to the activation of the two BGC for the known xenobactin (**11**)^20^ and szentiamide (**13**)^21^ for which the BGCs had not been identified yet (Supplementary Figure 3). Promoter exchange of Xsze_03663 and Xsze_0377 in the Δ*hfq* mutant resulted in the production of an oxidized diketopiperazine named szentirazine (**14**) and three lipopeptides (**15 a**–**c**) that represent shortened PAX‐peptides (Figure 2 d),^22^ none of which are detected in the wild type strain. The structures of **15 a**–**c** were solved by detailed MS‐MS analysis (Supplementary Figure 4). Szentirazine (**14**) was isolated from a large‐scale culture and its structure was solved by NMR spectroscopy (Supplementary Figures 5–9, Supplementary Table 2). Compared to standard non‐ribosomal peptide synthetases (NRPS), the bimodular NRPS involved in the production of **14** encodes an additional *N*‐terminal acyl‐CoA dehydrogenase (ACAD) domain^23^ that might introduce the double bond (Supplementary Figure 3).

When the approach was applied to additional *Xenorhabdus*‐Δ*hfq* and *Photorhabdus*‐Δ*hfq* strains several known (Supplementary Figure 10–12) and new NPs were readily identified showing its broad applicability: the new peptides silathride (**23**) and flesusides A and B (**24 a** and **24 b**) from *Photorhabdus* PB45.5‐Δ*hfq* (Figure 2 d, Supplementary Figure 12, Supplementary Table 3) and the new lipopeptide cuidadopeptide (**25**) from *Xenorhabdus* KJ12.1‐Δ*hfq* (Figure 2 d). The structures of all new NPs were solved via a combination of labeling experiments and detailed mass spectrometry, including fragmentation analysis and comparison between the natural and synthetic NPs as shown for **23** and **24** (Supplementary Figure 13) and **25** (Supplementary Figures 14–16).

### Bioactivity testing of single‐NP‐enriched Δhfq culture supernatants

**Figure 3:**
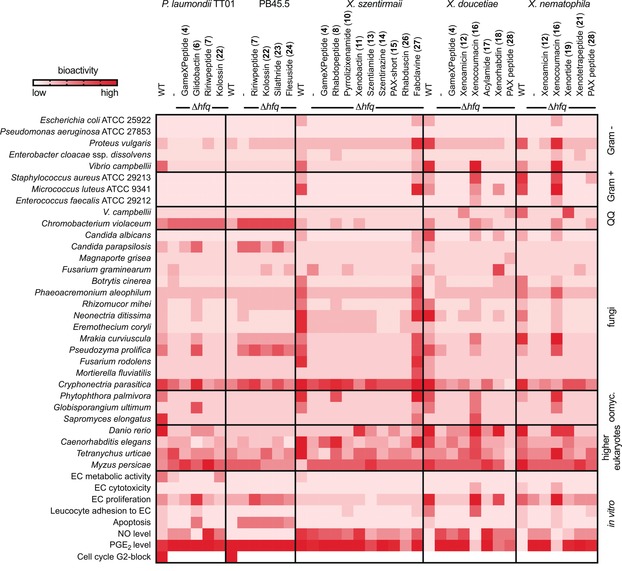
Bioactivity of cell‐free culture supernatants enriched in desired NPs (top) derived from Δ*hfq*‐P_*BAD*_‐xy mutants against wild type (WT) or Δ*hfq* alone against different organisms and in vitro assays. Bioactivities are shown for none (white) to highest activity (red) in the different assays (Supplementary Table 5). For NP data and structures see Supplementary Figure 1 and Supplementary Table 1.

Culture supernatants of induced Δ*hfq*‐P_*BAD*__xy mutants grown in Luria Bertani medium enriched only with the desired NP suggested the possibility of direct testing for bioactivity. We therefore tested 38 supernatants from different strains, including the corresponding wild type and Δ*hfq* controls, in multiple bioassays (Supplementary Table 5). These included antibiotic activity against Gram‐negative and Gram‐positive bacteria, quorum quenching (QQ) activity against *Vibrio campbellii* and *Chromobacterium violaceum*, activity against different human and plant pathogenic fungi, oomycetes, toxicity against higher organisms (zebrafish, nematodes, insects, mites) and biochemical assays (Figure 3).

In these assays we observed a loss of activity for all Δ*hfq* mutants compared to most wild type strains (Figure 3). One exception was an unknown quorum quenching activity in all Δ*hfq* mutants of both *Photorhabdus* strains. Several known bioactivities were confirmed with our method, including quorum quenching activity of the phenylethylamides (**17**) and tryptamides against *C. violaceum* 24 and apoptosis‐inducing activity of the proteasome inhibitor glidobactin A (**6**).^25^, ^26^ Glidobactin A (**6**) additionally showed antifungal activity and inhibited the production of NO, but not of prostaglandin E_2_ (PGE_2_) in vitro. Xenocoumacins (**16 a** and/or **16 b**)^27^ and fabclavine (**27**)^28^ appeared to be the main bioactive contributors in both *X. doucetiae* and *X. nematophila*. While xenocoumacins show a broad‐spectrum bioactivity in most assays including inhibition of NO and PGE_2_ production, fabclavines show a similar broad‐spectrum activity without inhibiting the production of NO and PGE_2_. It must be mentioned that from the activation of some BGCs, multiple NP derivatives are produced (e.g. for the rhabdopeptides, xenoamicins or GameXPeptides)^19^ and that the corresponding bioactivity data cannot identify the active derivative. However, once a desired bioactivity is observed, the most active derivative can be identified following isolation of these derivatives and repeating the target assay(s) with the pure NPs. Differences in the amount or structure of these derivatives might also account for bioactivity differences as it was observed for activation of the xenocoumacin producing BGC in *X. doucetiae* and *X. nematophila* (compare the production of **16** in Supplementary Figure 10 and 11). While both xenocoumacin I (**16 a**) and xenocoumacin II (**16 b**) are produced in wild type and promoter exchange mutant of *X. nematophila*, in *X. doucetiae* only **16 b** was observed but at a higher amount. Wild type supernatants of *P. laumondii* TTO1 showed a good antibiotic activity against *V. campbelli* that could not be repeated in any of the promoter exchange mutants suggesting that the responsible BGC was not activated. Activation of different BGCs in the same parental strain showed high bioactivity against higher eukaryotes like zebrafish and nematodes exemplified by **16**/**18** and **16**/**19** in *X. doucetiae* and *X. nematophila*. In general, the bioactivity of two NPs in the same assay might point towards an important ecological role of these NPs to act synergistically in a well‐defined (or concerted) mixture. The free‐living stage of the nematodes carrying *Photorhabdus* or *Xenorhabdus* in their gut, infect insect larvae in the soil that are used as a food source and shelter for nematode development. To guarantee an undisturbed propagation, the insect cadavers must be protected by NPs delivered by the nematode symbiont against potential food competitors including also invertebrates and vertebrates.^19^ Since many NPs are only produced in low amounts in the natural environment,^29^ synergism might be an efficient way to potentiate the overall activity as previously shown also for clinically used drugs^30^ while at the same time using less resources for NP production. Subsequently, activation of two BGCs together or mixing of the individual supernatants might help to elucidate such synergistic pairs.

### *easyPACId in* Pseudomonas entomophila

Since Hfq has been shown to influence the production of some NPs in other proteobacteria like *Pseudomonas,* 17, 31, 32 *Serratia* 33, 34, 35 and *Burkholderia,* 36 we applied our method to *Pseudomonas* (*Ps*) *entomophila* since it contained known and unknown BGCs. The Δ*hfq* mutant in *Ps. entomophila* showed a strong reduction of pyoverdines^37^ and labradorins (**30**)^38^ and a complete loss of the lipopeptide entolysin (**31**).^39^ *Ps. entomophila*‐Δ*hfq* lost its swarming ability and antibiotic activity against *Micrococcus luteus* and *Saccharomyces cerevisiae* (Supplementary Figure 17). Activation of the BGC (PSEEN_RS10885) for the recently described pyreudiones (**29**)^40^ indeed resulted in the products that were not produced in the wild type strain under the cultivation conditions tested (Supplementary Figure 17). Additionally, a new tetrapeptide named pseudotetratide A (**33**) (Figure 2 d) was obtained from activation of PSEEN_RS12600 in the Δ*hfq* mutant showing the potential of this approach in other NP‐producing proteobacteria encoding Hfq. The structure of **33** was confirmed after isolation from a large‐scale culture followed by detailed NMR analysis (Supplementary Figure 18–23, Supplementary Table 4).

### Chances and limitations of easyPACId

If BGCs are composed of multiple separated transcription units, promoter activation of only one of these would result in only partial BGC activation and production of either none or not the complete NP. To achieve full BGC activation for complete NP production, multiple promoters must be activated. This limitation also applies to promoter activation in Δ*hfq* mutants as it was evident for the BGC responsible for entolysin (**31**) biosynthesis in *Ps. entomophila* that is split into two loci *etlA* and *etlBC*.^39^ Activation of *etlA* encoding two NRPS modules only produced the starter fragments (**32 a**–**b**) of entolysin that were not detected in the wild type under the same conditions (Supplementary Figure 17). In cases where the biosynthesis of an unusual building block (e.g. amino acid, iso‐fatty acid) is also Hfq‐dependent, either no NP or non‐native NP derivatives will be produced which can also be advantageous due to the production of novel derivatives that the wild type does not produce. This is exemplarily shown for the production of xenorhabdins (**18**) in promoter exchange mutants of the *X. doucetiae* wild type and Δ*hfq* strains: neither an iso‐fatty acid nor an *N*‐methyl group was found in the xenorhabdins produced in the Δ*hfq* mutant in contrast to the derivatives produced in the wild type strain (Supplementary Figure 24).^10^ This suggests that enzymes responsible for these pathways/modifications are not encoded in the activated operon but encoded elsewhere in the genome and therefore are not produced in the Δ*hfq* mutant. However, in general the BGC structure in proteobacteria is often rather simple compared to other prolific NP producers like actinobacteria, making them ideal targets for this approach that we termed easyPACId.

Even if Δ*hfq* mutants would have a growth defect compared to the parental wild type strain as observed for *Ps. aeruginosa* 17 and *Ps. entomophila* (Supplementary Figure 2), the cleaner background of the Δ*hfq* strain would still be advantageously for NP detection and isolation.

Currently, >30.000 BGCs from >24.000 unique bacterial strains are listed in the antiSMASH database,^41^ a repository for microbial genomes analyzed via antiSMASH. The major BGC types encode NRPS, polyketide synthases (PKS), terpene synthases and pathways involved in the production of ribosomally‐synthesized and post‐translationally modified peptides (RiPPs) but also >2000 “other” BGCs, that have not been assigned to known BGC classes so far. While Actinobacteria are still representing the majority in such databases, clearly Proteobacteria have a huge potential. In the antiSMASH database, several BGCs are found in *Photorhabdus* (>380), *Xenorhabdus* (>490), *Serratia* (>1200), *Vibrio* (>4000), *Burkholderia* (>11500), and *Pseudomonas* (>12 400) that all might be accessible to a promoter exchange in Δ*hfq* mutants as all these strains show a reduction or loss in NP production as described here or in the literature.^17^, ^32^, ^33^, ^34^, ^36^

## Conclusion

Although we applied easyPACId mainly to NRPS and NRPS/PKS‐derived NPs as they often represent the major NP classes, we assume that it also works for other BGC classes that are controlled by a single promoter as it is often the case in proteobacteria.^42^ Since the generation of Δ*hfq* mutants as well as the activation of BGCs of interest can easily be performed in high‐throughput in these (and other) strains, it should be possible to obtain multiple new NPs in the future. This will accelerate the identification of bioactive NPs for various applications from direct testing of supernatants or crude extracts without time‐consuming isolation (Figure 3). In more well‐established NP producers like *Actinobacteria,* in the future maybe other global regulatory mechanisms could be used. As an example, it has been shown in *Streptomyces* that *N*‐acetylglucosamine acts as a signal for the onset of development and as a global elicitor molecule for antibiotic production.^43^ This said, there might also be a global suppressor mechanism for NP production in these bacteria that would be equivalent to Δ*hfq* mutants in proteobacteria and that can be used for promoter exchange approaches even in BGCs with multiple transcriptional units applying CRISPR/Cas^24^ or similar technologies. In general, the isolation of NPs from such mutants with a reduced NP‐background or no NPs at all would be greatly simplified allowing the future illumination of the biosynthetic “dark matter” present in most microbes.^10^, ^44^

## Conflict of interest

The authors declare no conflict of interest.

## Supporting information

As a service to our authors and readers, this journal provides supporting information supplied by the authors. Such materials are peer reviewed and may be re‐organized for online delivery, but are not copy‐edited or typeset. Technical support issues arising from supporting information (other than missing files) should be addressed to the authors.

Supplementary
