# Supplementary material for: Promoter Activation in Δhfq Mutants as an Efficient Tool for Specialized Metabolite Production Enabling Direct Bioactivity Testing
Source: Angew Chem Int Ed Engl. 2019 Dec 12;58(52):18957–63. doi: 10.1002/anie.201910563 (PMC6972681; doi:10.1002/anie.201910563)
Supplement: Supplementary file 1 — Supplementary [file ANIE-58-18957-s001.pdf]

Angewandte Chemie
Eine Zeitschrift der Gesellschaft Deutscher Chemiker

Supporting Information

Promoter Activation in Δ*hfq* Mutants as an Efficient Tool for Specialized Metabolite Production Enabling Direct Bioactivity Testing

*Edna Bode, Antje K. Heinrich, Merle Hirschmann, Desalegne Abebew, Yan-Ni Shi, Tien Duy Vo, Frank Wesche, Yi-Ming Shi, Peter Grün, Svenja Simonyi, Nadine Keller, Yvonne Engel, Sebastian Wenski, Reuel Bennet, Sophie Beyer, Iris Bischoff, Anthony Buaya, Sophie Brandt, Ibrahim Cakmak, Harun Çimen, Simone Eckstein, Denia Frank, Robert Fürst, Martin Gand, Gerd Geisslinger, Selcuk Hazir, Marina Henke, Ralf Heermann, Virginie Lecaudey, Wilhelm Schäfer, Susanne Schiffmann, Anja Schüffler, Rebecca Schwenk, Marisa Skaljac, Eckhard Thines, Marco Thines, Thomas Ulshöfer, Andreas Vilcinskas, Thomas A. Wichelhaus, and Helge B. Bode**

anie_201910563_sm_miscellaneous_information.pdf

Supplementary information

A. Materials and methods 3

1. Cultivation of bacteria 3

2. Cloning Methods 4

2.1 Deletion of *hfq* 4

2.2 Analysis of growth of WT and Δ*hfq* mutants 6

2.3 Construction of promoter exchange mutants 6

3. Generation of extracts for activity tests 7

4. Analytical methods 8

5. Stereochemistry assignment 9

6. Bioactivity testing of extracts 9

6.1. Antimicrobial activity 9

6.2 Quorum-quenching activity assay 13

6.3. Bioassays against higher eukaryotes 14

6.3.1. Zebrafish (*Danio rerio*) 14

6.3.2. *Caenorhabditis elegans* 15

6.3.3. Mites (*Tetranychus urticae*) 15

6.3.4. Aphids (*Myzus persicae*) 16

6.4. Endothelial cells (EC) 17

6.5. In vitro assays 19

B. Supplementary Tables 22

Supplementary Table 1. NPs mentioned in this work. 22

Supplementary Table 2. NMR spectroscopic data for szentirazine (**14**). 24

Supplementary Table 3. MS data for all new NPs shown in Fig. 2d. 25

Supplementary Table 4. NMR spectroscopic data for pseudotetratide (**33**). 26

Supplementary Table 5. Assays and organisms used for bioactivity testing. 27

Supplementary Table 6. Wild type strains used in this work. 28

Supplementary Table 8. List of general plasmids used in this work. 28

Supplementary Table 9. List of general primers used in this work. 28

C. Supplementary Figures 29

Supplementary Fig. 1. Structures of all NPs involved in the study. 30

Supplementary Fig. 2. Comparison of growth of wild type and Δ*hfq* mutants. 31

Supplementary Fig. 3. New NRPS and BGCs identified in this work. 32

Supplementary Fig. 4. MS-MS analysis of **15a-c**. 33

Supplementary Fig. 5. Structure and key 2D NMR correlations of **14**. 34

Supplementary Fig. 6. ^{1}H NMR spectrum of compound **14**. 34
Supplementary Fig. 7. HSQC spectrum of compound **14**. 35
Supplementary Fig. 8. COSY spectrum of compound **14**. 35
Supplementary Fig. 9. HMBC spectrum of compound **14**. 36
Supplementary Fig. 10. HPLC/MS data of wt and Δ*hfq* mutant of *X. doucetiae.* 37
Supplementary Fig. 11. HPLC/MS data of *X. nematophila.* 38
Supplementary Fig. 12. HPLC/MS data of *Photorhabdus* PB45.5. 39
Supplementary Fig. 13. HPLC/MS analysis of peptides **23** and **24**. 40
Supplementary Fig. 14. HPLC/MS of *Xenorhabdus* KJ12.1 Δhfq_PBAD_02330. 41
Supplementary Fig. 15. MS data from *Xenorhabdus* KJ12.1 Δhfq_PBAD_02330. 42
Supplementary Fig. 16. MS-MS Fragmentation of **25a**. 43
Supplementary Fig. 17. HPLC/MS data of *Ps. entomophila.* 44
Supplementary Fig. 18. Structure and key 2D NMR correlations of **33**. 45
Supplementary Fig. 19. ^{1}H NMR spectrum of compound **33**. 45
Supplementary Fig. 20. ^{13}C NMR spectrum of compound **33**. 46
Supplementary Fig. 21. HSQC spectrum of compound **33**. 46
Supplementary Fig. 22. HMBC spectrum of compound **33**. 47
Supplementary Fig. 23. COSY spectrum of compound **33**. 47
Supplementary Fig. 24. HPLC/UV analysis of xenorhabdins. 48
References 49

A. Materials and methods

1. Cultivation of bacteria

All wild type strains are listed in Supplementary Table S6. Cultivation of *Photorhabdus* and *Xenorhabdus* strains was performed in LB medium on a rotary shaker or on LB-agar plates supplemented with antibiotics in appropriate concentrations (ampicillin 100 µg/ml, rifampicin 50 µg/ml, kanamycin 50 µg/ml, chloramphenicol 34 µg/ml) at 30 °C. Cultivation of production cultures of *Pseudomonas entomophila* was performed in 5 ml XPP medium at 30 °C, inoculated with a 12 h LB pre-culture, on a rotary shaker at 200 rpm. If necessary cultures were induced with 0.2 % L-arabinose.

medium			
LB	LB (1 L)	10 g<br>5 g<br>10 g<br>ad VE H_2O	Trypton<br>Yeast extract<br>NaCl
		15 g	Agar (optional)
XPPM	XPPM (1 L)	10 g<br>20 ml<br>20 ml<br>2 g<br>1 g<br>ad VE H_2O<br>autoclaved	Glycerol<br>M9 salt A<br>M9 salt B<br>L-amino acid mix<br>Sodium pyruvate
		2 ml<br>1 ml	Vitamin solution<br>Trace element solution
	M9 salt A (1 L)	350 g<br>100 g	K_2HPO_4<br>KH_2PO_4
	M9 salt B (1 L)	29.4 g<br>50 g<br>5 g	Sodium citrate<br>$(NH_4)_2SO_4$<br>$MgSO_4$
	Trace element solution (1 L)	40 mg<br>200 mg<br>10 mg<br>10 mg<br>10 mg<br>10 mg<br>ad VE H_2O	$ZnCl_2$<br>$FeCl_3$ x 6 H_2O<br>$CuCl_2$ x 2 H_2O<br>$MnCl_2$ x 4 H_2O<br>$Na_2B_4O_7$ x 10 H_2O<br>$(NH_4)_6Mo_7O_{24}$ x 4 H_2O
	Vitamine solution (1 L)	10 mg<br>6 mg<br>200 mg<br>1 g<br>1.2 g<br>100 mg<br>2.3 g<br>12 g<br>20 mg<br>ad VE H_2O	Folic acid<br>Biotin<br>p-Aminobenzoic acid<br>Thiamin hydrochloride<br>Pantothenic acid<br>Riboflavin<br>Nicotinic acid<br>Pyridoxin hydrochloride<br>Vitamin B12

	L-amino acid mix	2 g<br>2 g<br>2 g<br>2 g<br>2 g<br>2 g<br>2 g<br>2 g<br>2 g<br>2 g<br>2 g<br>2 g<br>2 g<br>2 g<br>2 g<br>2 g<br>2 g<br>2 g	L-alanine<br>L-arginine<br>L-aspartate<br>L-asparagine<br>L-cysteine<br>L-glutamate<br>L-glutamine<br>L-glycine<br>L-histidine<br>L-isoleucine<br>L-leucine<br>L-lysine<br>L-methionine<br>L-phenylalanine<br>L-proline<br>L-serine<br>L-threonine<br>L-tyrosine

2. Cloning Methods

2.1 Deletion of *hfq*

The deletion of *hfq* in PB45.5, *X. doucetiae*, *X. szentirmaii* and *X. nematophila* was performed as described [1].

Photorhabdus* PB45.5-Δ*hfq

For the amplification of the 1016 bp upstream fragment and the 979 bp downstream fragment for the construction of the deletion plasmid primers AHp312/AHp313 and AHp314/AHp315 were used.

The plasmid pDelta_PB455_*hfq* was assembled from the *Pst*I/*Bgl*II linearized vector pCKcipB and both PCR-products via Hot Fusion Cloning [2]. After the second homologous recombination loss of *hfq* was verified using genomic DNA as a template with AHp316/AHp317 and AHp318/AHp319 yielding different length PCR products for the WT and the desired deletion mutant (PB45.5 WT: 859 bp, PB45.5Δ*hfq*: 550 bp) and (PB45.5 WT: 2335 bp, PB45.5Δ*hfq*: 2026 bp).

***X. doucetiae*-Δhfq**

For the deletion of *hfq* in *X. doucetiae* an upstream fragment of 1010 bp and a downstream fragment of 881 bp was generated with primers DA_03-fw/DA_04-rv and DA_01-fw/DA_02-rv. The fragments were fused via PCR and subsequently cloned into pEB17 (restricted with *Pst*I and *Bgl*II) via Hot Fusion cloning [2]. The deletion plasmid pEB17_DSM17909Δhfq was transformed into *X. doucetiae* via conjugation. After the

second homologous recombination the deletion of *hfq* was verified using primers V_DA_05-fw/VPEB_290-rv and VP_DA_06-rv/VPEB_289-fw.

X. nematophila*-Δ*hfq

For the deletion of *hfq* in *X. nematophila* an upstream fragment was amplified with primers NNp13 and NNp14. The downstream fragment was generated with AHp158 and AHp159. The fragments were fused via PCR and subsequently cloned into pCKcipB (restricted with *Pst*I and *Bgl*II) via hot fusion cloning. The deletion plasmid pCKcipB_DeltaXNC1_0455 was transformed into *X. nematophila* via conjugation. After the second homologous recombination the deletion of *hfq* in *X. nematophila* was verified using primers delta_hfq__XNC1_mut_ver_fw2 and delta_hfq__XNC1_mut_ver_rev2.

X. szentirmaii*-Δ*hfq

For the deletion of *hfq* in *X. szentirmaii* an upstream fragment was generated with primers ΔXSZ_hfq_up_PstI-Gib_fw and ΔXSZ_hfq_up_Gib_rev. The downstream fragment was generated with ΔXSZ_hfq_do_Gib_fw and ΔXSZ_hfq_do_BglII-Gib_rev. The fragemnts were fused via PCR and subsequently cloned into pCK_cipB (linearized with *Pst*I and *Bgl*II) via Hot Fusion cloning. The deletion plasmid pCK_cipB_Δhfq_XSZ (pNN06) was transformed into *X. szentirmaii* via conjugation. After the second homologous recombination the deletion of *hfq* in *X. szentirmaii* was verified using primers delta_hfq__XSZ_mut_ver_fw2 and delta_hfq__XSZ_mut_ver_rev2.

Ps. entomophila*-Δ*hfq

For the deletion of *hfq* in *Ps. entomophila* an upstream fragment was generated with primers PEB_534-fw and PEB_535-rv. With primers PEB_536-fw and PEB_537-rv the downstream fragment was amplified. The fragments were fused via PCR and subsequently cloned into pEB17 digested with *Pst*I and *Bgl*II. The deletion plasmid pEB17_Pseen_Δhfq was transformed into *Ps. entomophila* via biparental conjugation as described before[3]. *Ps. entomophila*-Δ*hfq* mutants were verified by PCR using primers VPEB_538-fw and VPEB_539-rv.

2.2 Analysis of growth of wild type and Δ*hfq* mutants

For growth comparison of wild type and Δ*hfq* mutant strains, overnight cultures of all strains grown in LB medium were adjusted to OD_{600} of 0.1. For *Ps. entomophila* XPP medium was used. Cultivation was performed in 180 µl culture volume in GRE96ft microtiter plates (Greiner) in triplicates. Incubation was carried out for 22 hours in a Spark® TECAN reader at 30 °C with shaking at 510 rpm. Optical density was measured every 20 min.

2.3 Construction of promoter exchange mutants

The vectors for promoter exchange were constructed as described earlier [4]. Briefly, the first 600-800 base pairs of the target gene were amplified with the corresponding primers all listed in Supplementary Table 7. The resulting fragment was cloned into pCEP (Supplementary Table 8) behind the P_{BAD} promoter via following methods 1-5 (resulting plasmid constructs are listed in Supplementary Table 7):

Method 1: The vector pCEP-KM (Supplementary Table 8) was digested with *Nde*I and *Pst*I and assembled with the according insert via Hot Fusion Cloning [2].

Method 2: pCEP-KM was linearized with *Nde*I and *Xba*I and fused with PCR amplified fragments of the respective biosynthetic gene via Hot Fusion Cloning.

Method 3: A pCEP-KM backbone was generated with the oligonucleotides pCEP_fw_gib and pCEP_rv_gib and assembled with the according insert via Gibson assembly. This was done with the Gibson Assembly Kit (New England BioLabs) following the manufacturer's instructions.

Method 4: A pCEP-KM backbone was generated with the oligonucleotides pCEP_fw_gib and pCEP_rv_gib (Supplementary Table 9) and assembled with the according insert via Hot Fusion.

Method 5: The vector pCEP or pCEP-KM was linearized using *Nde*I and *Sac*I restriction enzymes. PCR products were restricted with *Nde*I and *Sac*I and ligated with the linearized vector.

Ligated constructs were transformed into *E. coli* S17-1λpir that were plated on selective LB-Agar with kanamycin 50 µg/ml. The resulting clones were analyzed via colony PCR with verification primers V-pCEP-fw and V-pDS132-rv (Supplementary Table 9). Positive clones were used for conjugation with the Δ*hfq* strains. Conjugation was performed as described [4]. Briefly, from overnight cultures of the Δ*hfq* strain (acceptor) and *E. coli* S17-1λpir mutant strain (donor) fresh LB media was inoculated. Cultures were grown to an OD_{600} of 0.6-1.0. One ml of each, donor and acceptor strain was collected and cells were washed in LB medium if necessary. After pelleting via centrifugation cells were resuspended in 400 µl LB medium. For mating procedure, acceptor and donor were combined in a ratio of 1:3 and dropped on LB agar plates. After incubation at 37°C for the first three hours, plates were shifted to 30°C and incubated for 21h. The cells were scraped off and resuspended in 2 ml LB medium. Different volumes of the cell suspension were spread on selective LB agar plates containing ampicillin 100 µg/ml and kanamycin 50 µg/ml for *Xenorhabdus* strains and *Ps. entomophila*. For *Photorhabdus* strains rifampicin 50 µg/ml and kanamycin 50 µg/ml were used. Cells were incubated at 30°C for 24-72 h. Potential clones were analyzed with V-pCEP-fw and the respective verification primer (Supplementary Table 7 and 9).

The conjugation of *Ps. entomophila* was performed as described in [3,5].

For the swarming assay for *P. entomophila*, 5 µl of 12 h LB-cultures were dropped on LB-plates (Agar 0.5%, w/v). The plates were incubated at 30 °C for 12 h. Swarming behavior was determined after 24 h.

3. Generation of extracts for activity tests

All production cultures were cultivated in 1 l Erlenmeyer flasks containing 100 ml Luria-Bertani (LB) broth (pH 7.0) + 0,2% L-arabinose and inoculated with a 24 h pre-culture (0.1 %, v/v). For the pre-culture appropriate antibiotics were added to LB liquid when necessary at following concentrations: kanamycin 50 µg/ml and chloramphenicol 34 µg/ml. The cultures were cultivated at 30°C for 48 h and 140 rpm on a rotary shaker. The culture supernatant was separated from the cells by centrifugation for 20 min at 4000 rpm in an Eppendorf centrifuge in 50 ml Falkon tubes. 100 ml supernatant was lyophilized. The lyophilisate was dissolved in 10 ml deionised sterile water. The obtained 10-fold concentrated supernatants were either used directly or diluted according to the assay performed.

4. Analytical methods

HPLC/MS analysis of extracts, MS-MS fragmentation analysis and labelling experiments for MS-based structure elucidation of new NPs were all performed as described previously [6].

^{1}H, ^{13}C, HSQC, HMBC, ^{1}H-^{1}H COSY, and ROESY spectra were measured on a Bruker AV500 spectrometer, using DMSO as solvent. Coupling constants are expressed in Hz and chemical shifts are given on a ppm scale. HRESIMS was performed on an UltiMate 3000 system (Thermo Fisher) coupled to an Impact II qTof mass spectrometer (Bruker Daltonik GmbH). Column chromatography was performed with sephadex LH-20 (25–100 μm, Pharmacia Fine Chemical Co. Ltd.). Preparative HPLC was performed on an Agilent 1260 liquid chromatograph with a ZORBRAX StableBond 300 C18 (30 mm × 250 mm, 7.0μm, Agilent). Semi-preparative HPLC was performed on an Agilent 1260 liquid chromatograph with a ZORBRAX StableBond 300 C18 (9.4 mm × 250 mm, 5.0μm, Agilent).

Isolation of szentirazine (**13**) and pseudotetrapeptide (**33**) was performed as follows: *X. szentirmaii ∆hfq* pCEP-KM-1979 (producer of **13**) was cultured in LB (6L) with 2% XAD® resin at 30 ºC for three days. The resin was extracted exhaustively with methanol (3 × 2 L) at room temperature. The methanol extract was concentrated under reduced pressure to give 3.4g of a brown gum.

For isolation of pseudotetratide (**33**), *Ps. entomophila*Δ*hfq*_P_{BAD}#7 was cultivated in XPP medium (6 x 1L) with addition of 2 % XAD-16® resin. The media were inoculated with 10 mL 12 h LB –culture of *Ps. entomophila*Δ*hfq*_P_{BAD}#7 and induced with 0.2 % L-arabinose. The cultures were cultivated for 48 h at 30 °C on a rotary shaker with 130 rpm. Repeated extraction of the XAD-bound crude extract was performed with methanol (3 L in total). The organic phase was concentrated under reduced pressure to give 318.2 mg brown extract.

The residue (3.4 g) of cultivated *X. szentirmaii ∆hfq* pCEP-KM-1979 was subjected to Sephadex LH-20 (eluted with MeOH) to obtain eight fractions 1-8. Fraction 7 (58 mg) was separated by using semi-preparative HPLC with a gradient mobile from 20% to 33% ACN in H_2O (v/v) in 30 min to yield **1** (2.4 mg). The crude extraction (318.2 mg) *P. entomophila*Δ*hfq*_P_{BAD}#7 was separated by using preparative HPLC with a gradient mobile from 5% to 40% ACN in H_2O (v/v) in 30 min and further purified by semi-

preparative HPLC with a gradient from 10% to 30% ACN in H_2O (v/v) in 25 min to yield **2** (3.1 mg). Both of the ACN and H_2O used contained 0.1% formic acid.

5. Stereochemistry assignment

Compound **14** had a molecular formula of $C_{16}H_{15}N_5O_4$, as deduced from HR-ESI-MS at *m*/*z* 340.1056 $[M-H]^-$ (calcd for $C_{16}H_{14}N_5O_4$, 340.1051). By careful comparison, part of compound **14** was similar with dipodazine[7] (Supplementary Table 2). Combined with HMBC spectrum (from δ_H 11.66 to δ_C 136.1 and 127.5), the exchangeable signal at δ 11.66 was attributed to an indole NH. The linkage of the side chain was elucidated by ^1H-^1H COSY (Supplementary Fig. 1.) correlations of NH-1 (δ_H 8.22)/H-6 (δ_H 4.44)/H-7 (δ_H 2.85). The geometry of double bond was elucidated as *Z* configuration by the ROESY correlation of H-1' (δ_H 7.00) and H-2'' (δ_H 7.96). The asparagine residue was assumed to be L since the condensation (C) domain in xsze_1979 was annotated as ^LC_L and no epimerization (E) domain was present in the encoded NRPS.

Since **15** is produced by a BGC which resembles the first two modules of PAX peptide producing NRPS.[8] the configurations of the hydroxy group on the lipid chain and the Lys of **15a-c** were assumed to be consistent with PAX peptides as *R*-OH and *L*-Lys, respectively.

The configurations of amino acid residues in compounds **23-25**, **31a/b**, **32a/b**, and **33** were predicted based on a detailed analysis of the C, E and/or dual C/E domains occurring in the corresponding NRPSs[9] using antiSMASH. [10]

6. Bioactivity testing of extracts

6.1. Antimicrobial activity

For bioactivity testing against *Candida parapsilopsis*, *Magnaporthe grisea* and *Fusarium graminearum* the extracts were dried under the clean bench, followed by a chemical precipitation of the extracts with 70% EtOH and evaporation of the solvent to dryness under the clean bench. The precipitates were dissolved in water. Of these extracts 20 µL (1/10) were added to 180 µL minimal media[11] or potato-dextrose-broth (PDB (Carl Roth), containing pathogens (*Candida parapsilosis* OD_{600} 0.1; 8 conidia/µL *Magnaporthe grisea*; 1 conidia/µL *Fusarium graminearum*). About every 12 hours the growth was measured with a plate reader (Multimode Reader Mithras² LB 943 - Berthold Technologies). For *Candida parapsilosis* the absorption at OD_{600} was measured and for the other pathogens (*Magnaporthe grisea* and *Fusarium*

graminearum) the constitutive expressed **Green fluorescent protein** - GFP - signal (Absorption 460x10, Emission 520xm 10 UV) was used. Each pathogen was used in biological triplicates, while each measurement was performed in triplicates. As controls only 20 µL water instead of extracts.

For bioactivity testing against *Mrakia curviuscula* (Basidiomycota, Agaricomycotina, Tremellomycetes, Cryptobasidiales), *Pseudozyma prolifica* CBS 319.87 (Basidiomycota, Ustilaginomycotina, Ustilaginomycetes, Ustilaginales), *Globisporangium ultimum* Lev1805 (Oomycota, Peronosporomycotina, Peronosporomycetes, Pythiales), *Phytophthora palmivora* P16830 (Oomycota, Peronosporomycotina, Peronosporomycetes, Peronosporales), *Sapromyces elongatus* CBS 213.82 (Oomycota, Peronosporomycotina, Peronosporomycetes, Rhipidiales), *Fusarium rodolens* SBiKF 001.17 (Ascomycota, Pezizomycotina, Sordariomycetes, Hypocreales), and *Mortierella fluviatilis* SBiKF 002.17 (Zygomycota, Mucuromycotina, Mortiarellomycetes, Mortiarellales), agar diffusion assays were performed using 30 µl of each extract dropped onto a sterile 9 mm assay disc and air dried at ambient temperature to constitute a test-disc. Isolates were first grown in a 60 mm Petri plate on Potato Dextrose Agar (PDA, Roth, Germany), and incubated at room temperature prior to the assay for 5 – 7 days.

For the assay, *Mr. curvuiscula* and *Ps. prolifica* cell-suspension were done and adjusted to an absorbance of 0.1 at 600 nm (Implen Nanophotometer, Implen GmbH Munich, Germany). These cell-suspensions were spread onto a new 90 mm PDA plate using a Drigalski spatula, and test-discs were placed equidistantly onto the spread-plated PDA plates. For the mycelial cultures a 5 mm plug from the 5 – 7 day-old cultures of *G. ultimum*, *Ph. palmoviora*, *S. elongatus*, *F. rodolens*, and *Mo. fluviatilis* where placed in the centre of a new PDA plate and were allowed to grow for 2 – 3 days before placing the test discs equidistantly onto the test plates. Due to the difference in mycelial growth, inhibition zones were scored at different time-points after inoculation. The zone of inhibition (ZOI) as assessed from the rim of the test discs was measured after 24 h for *Ps. prolifica* and *G. ultimum*, after 36 h for *Mr. curviuscula* and *Mo. fluviatilis*, and 48 h for *F. rodolens*, *Ph. palmivora*, and *S. elongatus* and scored in five different categories.

Chestnut blight *Cryphonectria parasitica* was isolated from infected sweet chestnut trees in Aydin province of Turkey. Fungus culture was provided by Aydin Adnan

Menderes University, Faculty of Agriculture, Department of Plant Protection and maintained on sabouraud dextrose agar (SDA) at 28 °C. Extracts were incorporated into SDA at 3% (v/v). The amount of prescribed distilled water was reduced by 3 % to allow for subsequent addition of treatment suspensions. When the media cooled to 45-50 °C, 3 % extract was added and mixed thoroughly before pouring in 35 mm petri dishes. A 5 mm diameter mycelia plug was cut from margin of actively growing a fungal culture and placed on the center surface of each agar dish containing 3 % metabolite [12]. Metabolite free SDA plates were used as negative control and *Xenorhabdus szentirmaii* cell-free supernatant was used as positive control with the same concentration of the metabolites. Plates were incubated at 28°C and colony diameter was measured after 7 days. The resulting vegetative growth was recorded by using a ruler to measure the diameter of the colony (cm); two perpendicular measurements were made on each plate [13] In vegetative growth assay, five replicate dishes were used for each treatment.

For antibiotic assays against *Rhizomucor miehei* (Tü 284; 37°C)*, Eremothecium coryli* (ATCC 10647; 27°C)*, Candida albicans* (ATCC 381; 37°C), *Enterobacter cloaceae subsp. dissolvens* (LMG 2683; 27°C), and *Proteus vulgaris* (DSM 30119, 37°C), all fungi (except *E. lata*) were maintained on HA agar (per liter: 4 g glucose, 4 g yeast extract, 10 g malt extract, 2% agar; pH 5.5) and all bacteria on NB agar (per liter: 8 g Bacto-Nutrient broth, 1g NaCl, 2 % agar; pH 6.5). *E. lata* was cultured on BAF agar (per liter: 1 g yeast extract; 10 g glucose; 20 g maltose; 2 g peptone; 0.5 g KH_2PO_4; 0,4 g $MgSO_4x7H_2O$; 70 mg $CaCl_2x2H_2O$; 10 mg $FeCl_3x6H_2O$; 2 mg $ZnSO_4$, 2% agar; pH 5.5). Test compounds were added to 96-well-plates and solvents were evaporated under sterile conditions. As negative control the corresponding solvent was used. For positive control ciclopirox (for fungi) and tetracycline or streptomycine (for bacteria) were used. Before testing filamentous fungi (*R. miehei*) were inoculated on agar plates for sporulation for 3-4 days. Spores were obtained by adding HA medium to the plates and dispensing of spores by scratching. At $1x10^5$ spores/ml cultures were incubated at the appropriate temperature for 3 to 4 h at 120 rpm before 200 µl were added to the 96-well-plate with test compounds. Since the conidia of *E. lata* germinate poorly, 1 mg/ml wet weight of mycelium of a preculture (2 weeks old in BAF medium) was used for testing. One day before testing of *Er. coryli, C. albicans, En. cloaceae* and *P. vulgaris,* fluid cultures were inoculated and cultivated at the appropriate temperature

at 120 rpm. Approximately 16 h after inoculation cells were counted and diluted to $1x10^5$ cells/ml in medium. 200 µl of each suspension was added to the corresponding 96-well-plate with extracts. The inoculated 96-well-plates were incubated at the appropriate temperature for 16 to 24 h or for 48 h for *E. lata* at 120 rpm. Cell viability was assessed by eye. For *E. lata* ++ corresponds to 100% growth inhibition, + to 50-75%, and – to 0%. For *R. miehei, C. albicans, Er. coryli, En. cloaceae* and *P. vulgaris* + is equal to75-100% growth inhibition, +/- to 50-75%, and – to 0%.

For the germination assays *Botrytis cinerea* (growth at room temperature), *Ph. aleophilum* (*Togninia minima*, CBS 100398, 27°C), and *Neonectria ditissima* were used. *B. cinerea* was maintained and grown on 2% malt agar (per liter: 20 g malt extract, 2% agar; pH 5.5) for 3 to 4 weeks. *Ph. aleophilum* and *N. ditissima* were maintained on YMG agar (per liter: 4 g/L yeast extract, 10 g/L malt extract, 10 g/L glucose; pH 6.5) for 3 to 6 weeks. Test compounds were added to 96-well-plates and solvents were evaporated under sterile conditions. As negative control the corresponding solvent was used. For positive control ciclopirox was used. Spores were obtained by adding sterile water to the plates and dispensing of spores by scratching for *B. cinerea* and *Ph. aleophilum*. This suspension was filtered through Miracloth and centrifuged (2700 g; 20°C; 10 min). Spore concentration was adjusted to $2x10^6$ spores/ml in sterile water for *B. cinerea* and *Ph. aleophilum*. Spores of *N. ditissima* were obtained by inoculating 1L flasks with one baffle filled with 500 ml AM medium (Apple agar (AM) was prepared by using two cored and shredded Breaburn apple which were autoclaved in 1 L deionized water, this mixture was subsequently filtered, 5 g of glucose were added to the flow through, the volume filled up to 1 L, and autoclaved again to give AM). Cultivation took 10-14 days at 22-25°C at 150 rpm until spores were seen. Spores were prepared by filtration through sterile Miracloth and centrifugation (2700 g; 20°C; 10 min). Spore concentration for *N. ditissima* was adjusted to $3.5x10^6$ spores/ml in sterile water. 2% malt medium or YMG medium was added to the 96-well-plate with test compounds for *B. cinerea*, or *Ph. aleophilum* and *N. ditissima,* respectively. Spore suspension was added to the test plates (final spore concentration: $1x10^5$ spores/ml for *B. cinerea*, or *Ph. aleophilum* and $3.5x10^6$ spores/ml for *N. ditissima*). The inoculated 96-well-plates were incubated at the appropriate temperature for 16 to 24 h or 48 h for *Ph. aleophilum* ad *N. ditissima*. Viability and spore germination were evaluated by eye. For *B. cinerea* and *N. ditissima* +++

corresponds to 100% growth/germination inhibition, ++ to 75%, + to 50% and – to 0%. For *Ph. aleophilum* + is equal to75-100% growth/germination inhibition, +/- to 50-75%, and – to 0%.

For bioactivity against *Stapylococcus aureus* ATCC 29213, *Enterococcus faecalis* ATCC 29212, *Escherichia coli* ATCC 25922, *Pseudomonas aeruginosa* ATCC 27853, *Micrococcus luteus* ATCC 9341 bacteria were suspended in sterile saline to the density of a McFarland 0.5 standard, corresponding to 1-2 x 10^8 CFU/ml and the inoculum was spread on Mueller Hinton agar (Oxoid, Wesel, Germany) by a sterile cotton swab according to EUCAST guidelines [14]. Discs (Macherey-Nagel, Düren, Germany) with a diameter of 6 mm were loaded with 10 µl of extracts and placed on the agar. After incubation at 36°C for 16-20h the zones of inhibition were determined.

For antibiotic activity against *Chromobacterium violaceum* and *Vibrio campbellii* strains were grown at 30° C in LB and LM medium, respectively. 20 µl of the respective natural compound was pipetted into each well of a 96-microtiter plate. An overnight culture of the respective strain was diluted to an OD_{600}= 0.2 and a volume of 150 µl added to each well. OD was monitored every hour with a Sunrise plate reader (Tecan, Crailsheim).

6.2 Quorum-quenching activity assay

For testing on quorum quenching activity, we used *Chromobacterium violaceum* and *Vibrio campbellii* BB120 as reporter strains. At first, inhibition of quorum-sensing dependent violacein production of *C. violaceum* was measured. For that purpose, *C. violaceum* wild-type was grown at 30° C in LB medium [1% (w/v) NaCl; 1% (w/v) tryptone; 0.5% (w/v) yeast extract]. An overnight culture of *C. violaceum* with an OD_{600}=2-3 was added to liquid hand-warm LB soft-agar 0.8% (w/v) in dilution of 1:100. Antimicrobial susceptibility disks with 20 µl of the respective natural compound were dropped onto the solidified plates which were then incubated at 30°C overnight. On the next day the halo diameter around the antibiotic-testplate was measured.

As a second read-out to analyze AHL-degrading activity of the natural compounds produced by *Photorhabdus* and *Xhenorhabdus* species quorum-sensing dependent bioluminescence of *V. campbellii* wild-type was used. Therefore*, V. campbellii* was grown at 30° C in LM medium [10% (w/v) peptone, 5% (w/v) yeast extract, 20% (w/v)

NaCl] supplemented with carbenicillin (100 µg/ml). An overnight culture of *V. campbellii* was inoculated at OD_{600}=0.2 in LM and a volume of 150 µl was pipetted into each well of a 96-well microtiter plate. Cells were grown aerobically at 30° C for 2 hours and then a volume of 20 µl of the respective natural compound was added to each well. OD and luminescence were monitored every hour with a Sunrise plate reader (Tecan, Crailsheim) and a Centro luminometer (Berthold Technologies, Bad Wildbad), respectively.

6.3. Bioassays against higher eukaryotes

6.3.1. Zebrafish (*Danio rerio*)

Wild type (Tü) zebrafish *Danio rerio* were maintained according to standard procedures and embryos were obtained by natural spawning (Westerfield, zebrafish book).

Procedures involving animals were conducted according to the guidelines of Goethe University in Frankfurt, Germany and approved by the German authorities (veterinary department of the Regional Board of Darmstadt).

Embryos were incubated in E3 (5.0 mM NaCl, 0.17mM KCl, 0.33mM CaCl2, 0.33mM MgSO4) at 28.5 °C at all times and placed in 24-well plates at a density of 20 embryos per well.

Diluted extracts were added at 2hours post-fertilization (hpf) and embryos were observed after 24h and 48h of treatment. Extracts were diluted 10 times in ddH20 except extracts 57-62, which were diluted 100 times in Methanol:Aceton 1:1. For this purpose 1 µL of extract was first diluted in 50µL of Aceton and 50µL of Methanol were then further added. All extracts were thoroughly vortexed before use. The Fibroblast Growth Factor Receptor (FGFR)-inhibitor SU5402 (20 µM in DMSO) was used as positive controls and ddH20 alone as negative controls. After 24h and 48h of treatments, the embryos were given a “toxicity score” between 1 and 5 based on the following criteria:

1: > 80% embryos normal or with only a slight developmental delay

2: > 80% embryos show strong developmental delay

3: > 80% embryos show strong malformation

4: > 80% embryos are dead

5: > 80% embryos fully degraded

6.3.2. *Caenorhabditis elegans*

Nematicidal activity was performed using L4-stage of *C. elegans* in 24-well microtiter plates [15], where cell-free supernatant of different bacterial strains was added for testing.

Culturing of *C. elegans* was performed under standard culturing conditions on nematode growth medium (NGM) agar plate [50mM NaCl, 0.25% (w/v) peptone, 1 mM $CaCl_2$, 5 µg/ml cholesterol, 25mM KH_2PO_4, 1mM $MgSO_4$, and 1.7%(w/v) agar]. Overnight culture of *E. coli* OP50 was seeded on NGM as a food source for nematode. The wild-type Bristol N2 strain of *C. elegans* was cultivated for three days at 25°C on the bacterial lawn. The plates were washed with Luria-Bertani (LB) liquid medium and filtered through sieve with pores of 40µm [16].

The bacteria to be tested were sub-cultured for two days at 30°C with 200 rpm shaking in LB liquid medium. The cell-free supernatants were prepared by centrifugation at 4000rpm for 30mn and filtered through 0.2µm filter.

In this assay, 80-100 L4-stage *C. elegans* were added in to a well of 24-well microtiter plate containing 300µl of cell-free supernatant of bacteria strains to be tested. These nematodes were incubated at 25°C and examined under dissecting microscope every 24h for three days. The cell-free supernatant of *E. coli* OP50 was used as a negative control. The killing assay was conducted in triplicates. The nematodes were classified as dead when no movement was observed under a dissecting microscope and when their bodies become straightened. Mortality of the nematodes was calculated as the ratio of dead nematodes over the total number of tested nematodes.

6.3.3. Mites (*Tetranychus urticae*)

Mass rearing of *Tetranychus urticae*

T. urticae was collected from strawberry fields in Aydin, Turkey and reared on bean plants in a room which had the same climate conditions where the host plant production was grown. Detached bean leaves (five or six) infested with different stages of *T. urticae* were transferred to the *T. urticae* rearing room. The rearing of *T. urticae* was performed continuously during the study using the method as described [17,18].

Bean plants (*Phaseolus vulgaris* cv. barbunia) were used for rearing and experiments of *T. urticae*. Plants were grown in climate-controlled room (25±2°C, 60±10% Rh, 16 h light) until the formation of first 5-6 leaves and then they were transferred to another room for the rearing of *T. urticae*.

Efficacy of bacterial metabolites against *T. urticae*

The efficacy of the extracts against *T. urticae* was assessed with Petri dish experiments. Moistened cotton was placed in each dish (9 cm diameter) and then a bean leaf was cut 6 cm diameter with scissor and placed bottom side facing up on the cotton. Twenty females obtained from the culture were transferred into each Petri dish with a fine hair brush (size 000). Different dilutions of extracts (50, 25, 12.5 %) were separately sprayed onto the leaf by a hand sprayer and a total volume of 0.3 ml of suspension was applied to each leaf surface. The number of living and dead individuals was recorded daily for four days. The same volume of sterile distilled water was sprayed on the leaf infested with mites as control. Four replicates were used for each treatment and the experiments were conducted three times on different dates. The extracts showing more than 50% mite mortality were selected for further dilution assessment. Otherwise, the metabolites causing less than 50% mortality were not tested at lower dilutions.

Data were analyzed using analysis of General Linear Model. Means were compared at the P=0.05 level, and Tukey's test was used to separate means. Arcsine transformation was performed on mite mortality before statistical analyses (SPSS, 2011).

6.3.4. Aphids (*Myzus persicae*)

Parthenogenetic clone of *Myzus persicae* was maintained on the turnip plant *Brassica rapa* L. subsp. (cv. Tonda A Colletto Viola) in a climate cabinet (KBWF 720, Binder GmbH, Tuttlingen, Germany) with a 16-h photoperiod, a day/night temperature of 24/18 °C and 60% relative humidity.

In this study, we tested the extracts for insecticidal activity against *M. persicae.* For each extract, a stock solution of 10-fold was prepared in acetone/methanol (1:1) and then working solutions were diluted with artificial AP3 diet (Febvay et al., 1988) for aphids. In order to investigate insecticidal potency of bacterial extracts against *M. persicae* we tested seven fourfold dilutions (4, 1, 0.250, 0.063, 0.016, 0.004, 0.001-fold) of each extract. Positive control was AP3 diet supplemented with the chemical insecticide imidacloprid (0.4 µg/mL) (Sigma-Aldrich, Taufkirchen, Germany), while mixture AP3 diet and acetone/methanol (1:1) was used as negative control. Imidacloprid is highly hydrophobic, and it was prepared first as a concentrated stock

(1000 µg/mL) in acetone and then working solution was diluted in the AP3 diet. Each treatment in this study was tested in three replicates.

A rapid aphid bioassays were carried out in 48-well plates covered with a thin layer of Parafilm M consisting of 48 sachets. Each well contained 10-15 aphids on a small excised leaf of *B. rapa*. Individuals of *M. persicae* could easily reach a sachet which contained 10 µL of AP3 diet supplemented with extract or controls, and feed by piercing Parafilm membrane. Bioassay plates were kept under controlled conditions, as described previously. Mortality was assessed after 3 days of aphid exposure to the test treatments in a way that mean value of survived aphids was counted from replicates of the negative control and mortality threshold was set to 70% in order to evaluate all other treatments. Bacterial extracts that showed mortality of 70% and higher in at least two replicates were considered as “active”, while those that had lower percent mortality were considered as “non-active” [19]. Survival of aphids was not affected in the negative control, while none of the aphids survived insecticide control treatment after 3 days of exposure (data not shown).

6.4. Endothelial cells (EC)

Cell culture

The human microvascular endothelial cell line CDC.EU.HMEC-1 was kindly provided by the Centers of Disease Control and Prevention (CDC, Atlanta, USA) [20]. HMEC-1 cells were cultivated on collagen G (Biochrom AG, Berlin, Germany) coated plastic ware in Endothelial Cell Growth Medium (ECGM, PELOBiotech) containing 10 % heat-inactivated fetal calf serum (FCS; Biochrom, Berlin, Germany), 100 U/ml penicillin (PAN-Biotech, Aidenbach, Germany), 100 µg/ml streptomycin (PAN-Biotech) and 0.25 µg/ml amphotericin B (PAN-Biotech). The human monocytic-like cell line THP-1 was originally established from the peripheral blood of a one-year-old boy suffering from acute monocytic leukemia (AML). THP-1 cells were cultured in RPMI-1640 medium (RPMI, PAN-Biotech) containing 10 % heat-inactivated FCS, 100 U/ml penicillin and 100 µg/ml streptomycin [PMID: 6970727]. Both cell lines were incubated at 37 °C in an atmosphere of 5% CO_2 and 95% air. HMEC-1 cells were used for experiments up to passage 20 and THP-1 cells up to passage 30 without altering their cell-type specific morphology and functional characteristics.

Cell viability assay

The effect of aqueous extracts on the metabolic activity of HMEC-1 were analyzed using a CellTiter-Blue cell viability assay (Promega GmbH, Mannheim, Germany). In brief, HMEC-1 cells were grown to confluence in 96-well plates and treated with the indicated extracts or vehicle control (1:100 dilution) for 24 h. The resazurin containing CellTiter-Blue reagent was added to the cells in a ratio of 1:10 for the last 4 h of treatment. Since viable cells are able to reduce resazurin into fluorescent resorufin, the metabolic activity was determined by fluorescence measurements (ex: 579 nm, em: 584 nm) using a microplate reader (Tecan Infinite F200 Pro, Tecan, Männedorf, Switzerland).

Determination of cell number

To investigate possible cytotoxic effects of the aqueous extracts on HMEC-1 cells, the number of cells was determined using crystal violet solution: HMEC-1 cells were grown to confluence in 96-well plates and treated with the indicated extracts or vehicle control (1:100 dilution) for 24 h or pre-treated with the indicated extracts or vehicle control for 30 min and activated with TNFα (10 ng/ml) for 24 h. Afterwards, cells were washed once with PBS containing Ca^{2+} and Mg^{2+} and fixed with a methanol-ethanol (ratio 2:1) solution for 10 min before staining with crystal violet solution (0.5 % in 20 % methanol, Sigma-Aldrich, Taufkirchen, Germany) for 15 min. After removing the unbound residual crystal violet solution with tab water, the cells were dried for at least 24 h. Crystal violet bound to DNA was resolved in 20 % acetic acid and absorption was measured at 590 nm using a microplate reader (Tecan).

Proliferation assay

The impact of the aqueous extracts on the proliferation of HMEC-1 cells was investigated by measuring the cell number after staining cells with crystal violet. In brief, 2 x 10^3 cells per well were seeded in 96-well plates. After 24 h the cells were treated with the indicated extracts or vehicle control in a dilution range starting from 1:10 and 1:100, continuing with a 1:1 dilution until the extracts ceased to affect cell viability or proliferation. After 72 h the number of cells was determined using crystal violet solution as described above, whereby the untreated control cells were fixed and stained directly 24 h after seeding. To determine effects of the extracts on the relative proliferation rate of HMEC-1 cells, the obtained data from untreated control cells (fixed

and stained after 24 h) were subtracted from values obtained from stimuli treated cells (fixed and stained after 72 h).

Cell adhesion assay

To analyze the influence of the aqueous extracts on the adhesion of the human monocytic-like cell line THP-1 onto HMEC-1 cells, cell adhesion assays were performed, in which untreated THP-1 cells were allowed to adhere to HMEC-1 cells treated with aqueous extracts: HMEC-1 cells were grown to confluence in 96-well plates and pre-treated with the indicated extracts or vehicle control (1:100 dilution) for 30 min and activated with TNFα (10 ng/ml) for 24 h. THP-1 cells were stained in serum-free RPMI with CellTracker Green (5 µM, Thermo Fisher Scientific, Waltham, MA, USA) for 30 min at 37 °C in a water bath. Afterwards, THP-1 cells were washed once with serum-free RPMI before the cell concentration was adjusted with serum-containing RPMI to 2 x 10^5 cells per ml. The culture medium was removed from the treated HMEC-1 cells and THP-1 cells (2 x 10^4 cells per well) were allowed to adhere for 5 min. Non-adherent cells were removed by washing with PBS containing Ca^{2+} and Mg^{2+} and the adhesion was quantified by fluorescence measurements (ex: 485 nm, em: 535 nm) using a microplate reader (Tecan). The measured adhesion levels were normalized to the number of HMEC-1 cells that were present after treatment with aqueous extracts, which was determined using crystal violet solution as described above.

6.5. In vitro assays

Cells and reagents

HCT116 cells were cultured in McCoy's 5A (Modified) medium supplemented with 10 % FBS and 1 % Pen/Strep. RAW264.7 macrophages were cultured in RPMI1640 medium supplemented with 10 % FBS and 1 % P/S. Lyophilized extracts were reconstituted with water to a 10x concentration. This stock solution was used to dilute the extracts to a final dilution of 1:10 in the respective media.

Apoptosis assay

20,000 HCT 116 cells were seeded in a black poly-D-lysine-coated 96-well plate and incubated for 24 h at 37 °C. The culture medium was replaced by 100 µl DMEM medium without phenol red (Gibco, 21063-029) supplemented with 10 % FBS and 1

% Pen/Strep and containing extracts diluted 1:10 or 100 µM celecoxib as positive control (data not shown). Cells were incubated for 24 h at 37 °C with 5% CO_2. One µl of CellEvent Caspase-3/7 Green Detection Reagent (1:10 diluted in DMEM-medium without phenol red) was added and incubated for 90 min at 37 °C (without CO_2). Afterwards, 1 µl of DRAQ5 (1:25 diluted in DMEM-medium without phenol red) was added and the cells were incubated for 30 min at RT. Images were acquired using the ImageXpress Micro Confocal High Content Imaging System (Molecular Device, San Jose, USA). The percentage of dead cells was determined using the "live/dead"-analysis tool from Molecular Device by calculating the ratio of apoptotic cells (Caspase-3/7 positive cells) to all cells (DRAQ5 positive cells). Four different measuring sites in the well were analyzed and the average value was used for data presentation. The amount of apoptotic cells was related to all cells to determine the apoptotic rate. To simplify the presentation of data the apoptotic rate were clustered in 10 groups. An apoptotic rate from 0-10 % was classified as 1, from 11-20 % as 2 and so on.

Cell cycle assay

20,000 HCT116 in 100 µl medium were seeded in a 96-well cell culture plate and incubated for 24 h at 37 °C. The culture medium was replaced by 100 µl medium containing extract diluted 1:10 or control substances. As negative control medium with 10% FBS was used. G1-block was induced by medium without 10 % FBS. 20 µM curcumin and 0.2 µM staurosporine were used to induce G2- and S-block, respectively (data not shown). After an incubation step of 24 h at 37 °C, cells were harvested, suspended in 200 µl sample buffer (1 g glucose/l PBS without calcium or magnesium), mixed, centrifuged (200 g, 4 min, 4 °C) and supernatant was discarded. This step was repeated once. The supernatant, apart from 10 µl was removed. The cell pellets were vigorously vortexed in the remaining buffer for 10 s (~750 rpm). With continuous shaking (~500 rpm), 150 µl of ice-cold 70 % ethanol were added drop by drop to the pellet. For maximum resolution of cellular DNA, the samples were allowed to fix in ethanol overnight (> 18h) at 4 °C. Samples were vortexed, suspended in 100 µl sample buffer, centrifuged (500 g; 10 min; 4 °C) and supernatants were removed. Cell pellets were resuspended and incubated for 40 min at room temperature with 100 µl staining buffer (20 µl/ml propidium iodide and 0.2 mg/ml RNase in sample buffer). Samples were vortexed and measured within 24 hours in a MACSQuant Analyzer (Miltenyi

Biotec GmbH, Bergisch Gladbach, Germany). Cell cycle distribution was determined using FlowJo software.

NO- and PGE_2 determination

20,000 RAW264.7 macrophages in 100 µl medium were seeded in a 96-well cell culture plate and incubated for 24 h at 37 °C. To test if the extracts induce NO and/or PGE_2 release the different extracts (diluted 1:10) were added. As positive control 100 ng/ml lipopolysaccharide (LPS) was used. To test if the extracts inhibit NO and/or PGE2 release the cells were pre-incubated with extracts for 1 h before LPS were added for the stimulation of NO and PGE_2 synthesis. After an incubation step of 24 h at 37 °C supernatants were collected and stored at -80°C. The PGE_2 concentration was determined using a PGE_2 ELISA (Enzo Life Sciences GmbH, Lörrach, Germany). The ELISA was achieved as recommended by the supplier. The NO concentration was determined using the NO assay developed by Griess [21]. Briefly, 80 µl cell supernatant or standard sample were added in a 96 well microplate and thereafter, 20 µl sulfanilamide solution (120 mg sulfanilamide in 30 ml 1N hydrochloric acid) und 20 µl naphtylendiamine solution (180 mg N-(naphtyl) ethylendiamine dihydrochloride in 30 ml water) were added. After an incubation step of 15 minutes at room temperature the absorbance at 540 nm was detected using the EnSpire® Multimode Plate Reader (Perkin Elmer, Hamburg, Germany). For NO/PGE_2 induction/inhibition results the released amount of NO/PGE_2 from samples was related to the LPS-value. Since all supernatatnts might contain at least traces of LPS only a clear reduction of NO level is obvious for some extracts. To simplify presentation of data the induction/inhibition rates were clustered in 10 groups. A rate of 0-10% was classified as 1, of 11-20 % as 2 and so on.

B. Supplementary Tables

Supplementary Table 1. NPs mentioned in this work. Structures are shown in Supplementary Fig.2. Double charged MS signals are marked (*).

Name	Compound	Detected *m/z* $[M+H]^+$	Reference
Anthraquinone (AQ-270b)	**1**	271.1	[22]
Isopropylstilbene (IPS)	**2**	255.1	[23,24]
Phurealipid-229 (PL-229)	**3**	229.3	[25]
GameXPeptide A (GXPA)	**4a**	586.4	[26,27]
GameXPeptide C	**4b**	552.4	
Photopyrone D (PPYD)	**5**	295.2	[28]
Glidobactin A	**6**	521.3	[29,30]
Ririwpeptide A	**7**	372.2*	[31]
Rhabdopeptide (RXP-645)	**8a**	645.4	[32,33]
Rhabdopeptide (RXP-659)	**8b**	659.4	
Rhabdopeptide (RXP-758)	**8c**	758.4	
Rhabdopeptide (RXP-772)	**8d**	772.4	
Rhabdopeptide (RXP-885)	**8e**	885.5	
Rhabdopeptide (RXP-574)	**8f**	574.4	
Rhabdopeptide (RXP-687)	**8g**	687.4	
Xenofuranone B	**9**	281.3	[34]
Pyrrolizixenamide	**10**	251.1	[35]
Xenobactin	**11**	756.4	[36]
Xenoamicin-650	**12a**	650.8*	[37]
Xenoamicin-657	**12b**	657.8*	
Xenoamicin-643	**12c**	643.8*	
Xenoamicin-630	**12d**	630.4*	
Xenoamicin-637	**12e**	637.4*	
Xenoamicin-644	**12f**	644.4*	
Xenoamicin-651	**12g**	651.4*	
Szentiamide	**13**	838.4	[38,39]
Szentirazine	**14**	340.2	this work
PAX-short	**15a**	428.3	this work
PAX-short	**15b**	430.3	
PAX-short	**15c**	456.3	

Xenocoumacin 1 (XCN1)	**16a**	466.2	[40,41]
Xenocoumacin 2 (XCN2)	**16b**	407.2	
Phenylethylamide-240 (PEA-240)	**17a**	240.3	[42]
PEA-248	**17b**	248.3	
Xenorhabdin-299 (XRD-299)	**18a**	299.1	[4,43]
XRD-315	**18b**	315.1	
Xenortide A	**19**	410.2	[44]
Nematophin	**20**	273.1	[45]
Xenotetrapeptide	**21**	411.2	[46]
Kolossin B	**22**	843.6*	[47]
Silalthride	**23**	546.8*	this work
Flesusid A	**24a**	627.8*	this work
Flesusid B	**24b**	620.4*	this work
Cuidadopeptide	**25**	625.4*	this work
Rhabduscin	**26**	333.3	[48]
Fabclavine IIa	**27**	1312.9	[49]
PAX-peptide	**28**	1050.8	[8]
Pyreudione A	**29a**	266.1	[50]
Pyreudione B	**29b**	294.1	
Labradorin A	**30a**	241.1	[51,52]
Labradorin B	**30b**	255.1	
Entolysin (Etl-870)	**31a**	870.0*	[53]
Entolysin (Etl-861)	**31b**	861.0*	
Entolysin-431	**32a**	431.5	this work
Entolysin-302	**32b**	302.2	this work
Pseudotetratide A	**33**	528.2	this work

Supplementary Table 2. ^{1}H (500 MHz) and ^{13}C (125 MHz) NMR spectroscopic data for szentirazine (**14**) in DMSO-d_6 (δ in ppm and J in Hz).

no.	**14**	
	δ_H (mult., J)	δ_C
1 (NH)	8.22 (s)	
2		161.1
3		123.1
4 (NH)		
5		166.5
6	4.44 (dt, 5.1, 2.5)	51.6
7	2.85 (brs)	38.9
8		171.8
9 (NH)		
10		165.4
11 (NH)	8.38 (s)	
1'	7.00 (s)	107.8
1'' (NH)	11.66 (s)	
2''	7.96 (d, 2.6)	126.8
3''	-	108.4
3''a		127.5
4''	7.65 (d, 7.9)	118.7
5''	7.11 (m)	120.4
6''	7.18 (m)	122.6
7''	7.43 (d, 8.0)	112.5
7''a		136.1

Supplementary Table 3. MS data for all new NPs shown in Fig. 2d.

NP	m/z detected			Sum formula
	$^{12}C^{14}N$	$^{12}C^{15}N$	$^{13}C^{14}N$	
**14**	340.1056			$C_{16}H_{14}N_5O_4$ [M-H]$^-$
**15a**	428.3130	431.3 (N_3)		$C_{22}H_{42}N_3O_5$ [M+H]$^+$
**15b**	430.3282	433.3 (N_3)		$C_{22}H_{44}N_3O_5$ [M+H]$^+$
**15c**	456.3442	459.3 (N_3)		$C_{24}H_{46}N_3O_5$ [M+H]$^+$
**23**	546.8368	552.3209 (N_{11})	572.9245 (C_{52})	$C_{52}H_{90}N_{11}O_{14}$ [M+H]$^+$
**24a**	627.8477		653.9358 (C_{52})	$C_{52}H_{91}N_{19}O_{17}$ [M+H]$^+$
**24b**	620.3609		646.9504 (C_{52})	$C_{53}H_{94}N_{18}O_{16}$ [M+H]$^+$
**25**	625.3992	631.3836 (N_{12})	657.5065 (C_{64})	$C_{64}H_{106}N_{12}O_{13}$ [M+H]$^+$
**32a**	431.2745			$C_{21}H_{39}N_2O_7$ [M+H]$^+$
**32b**	302.2321			$C_{16}H_{32}NO_4$ [M+H]$^+$
**33**	528.2012	535.1808 (N_7)	549.2714 (C_{21})	$C_{21}H_{34}N_7O_5S_2$ [M+H]$^+$

Supplementary Table 4. ^{1}H (500 MHz) and ^{13}C (125 MHz) NMR spectroscopic data for pseudotetratide (**33**) in DMSO-d_6 (δ in ppm and J in Hz).

no.	**33**	
	δ_H (mult., J)	δ_C
1	1.82 (s)	22.8
2		169.7
3	8.15 (d, 8.32)	
4	4.55 (m)	50.9
5	1.90 (overlap)	28.9
	1.49 (m)	
6	1.55 (m)	26.0
	1.42 (m)	
7	3.12 (m)	41.2
9		157.6
10		170.2
12	5.31 (dd, 7.9, 3.6)	58.3
13	2.29 (m)	33.3
	1.90 (m)	
14	1.98 (overlap)	24.3
15	3.77 (m)	47.2
16		172.4
18	8.11 (s)	123.4
19		149.4
21		160.0
22	8.40 (d, 6.4)	
23	4.05 (m)	24.3
24	1.98 (m)	33.3
25	2.45 (m)	30.4
27	2.02 (s)	15.1
28		175.2

Supplementary Table 5. Assays and organisms used for bioactivity testing of culture supernatants.

Assay	Organism/assay
Gram-negative	*Escherichia coli* ATCC 29522
	Pseudomonas aeruginosa ATCC 27853
	Proteus vulgaris
	Enterobacter dissolvens
	Vibrio campbelli
Gram-positive	*Staphylococcus aureus* ATCC 29213
	Micrococcus luteus ATCC 9341
	Enterococcus faecalis ATCC 29212
Quorum quenching	*Vibrio campbellii*
	Chromobacterium violaceum
Fungi	*Candida albicans*
	Candida parapsilosis
	Magnaporte grisea
	Fusarium graminearum
	Botrytis cinerea
	Phaeoacremonium aleophilium
	Mucur mihei
	Neonectria ditissima
	Nematospora coryii
	Mrakia curviuscula
	Pseudozyma prolifica
	Fusarium rodolens
	Mortierelia fluviatilis
	Cryphonectria parasitica
Oomycetes	*Phytophthora palmivora*
	Globisporangium ultimum
	Sapromyces elongatus
Higher eukaryotes	*Danio rerio* (zebrafish)
	Caenorhabditis elegans (nematode)
	Tetranychus urticae (mite)
	Myzus persicae (aphid)
In vitro assays	Endothelial cell (EC) metabolic activity
	EC cytotoxicity
	EC proliferation
	Leucocyte adhesion to EC
	Apoptosis against HCT116 cells
	Induction of NO
	Induction of Prostaglandin E_2
	Cell cycle analysis (G2-block)

Supplementary Table 6. Wild type strains used in this work.

Strain	Genotype	Reference
E. coli S17-1λpir	Tpr Smr recA thi rsdRM+ RP4::2-Tc::Mu::KM Tn7, λ pir phage lysogen	Invitrogen
Photorhabdus laumondii TT01	wild type	[54]
Photorhabdus PB45.5	wild type	[55]
Xenorhabdus doucetiae DSM17909	wild type	[56]
Xenorhabdus nematophila HGB081	wild type	[57]
Xenorhabdus szentirmaii DSM16338	wild type	[58]
Pseudomonas entomophila	wild type	[59]

Supplementary Table 7. List of Δ*hfq* mutants, promoter exchange mutants and primers and plasmids used for their construction.

See separate Excel file.

Supplementary Table 8. List of general plasmids used in this work.

Plasmid	Genotype	Reference
pCEP_kan	pDS132 based, R6K ori, oriT, kan^R, *araC*, P_{BAD}	[4]
pCEP	pDS132 based, R6K ori, oriT, cm^R, *araC*, P_{BAD}	[4]
pCK_cipB	pDS132 based, R6K ori, oriT, cm^R, *cipB*, *sacB*	[27]
pEB17	pDS132 based, R6K ori, oriT, kan^R, *cipB*, *sacB*	[42]

Supplementary Table 9. List of general primers used in this work.

Oligonucleotide	Sequence (5´-3´)
VpDS132-fw	GATCGATCCTCTAGAGTCGACCT
VpDS132-rv	ACATGTGGAATTGTGAGCGG
VpCEP-fw	GCTATGCCATAGCATTTTTATCCATAAG
pCEP-BB-fw-gib	ATGTGCATGCTCGAGCTC
pCEP-BB-rv-gib	ATGCTAGCCTCCTGTTAGC

C. Supplementary Figures

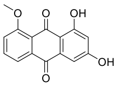
1

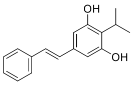
2

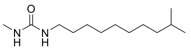
3

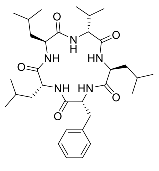
4a

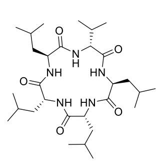
4b

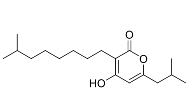
5

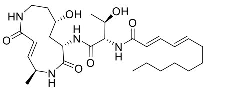
6

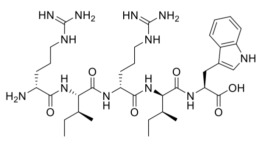
7

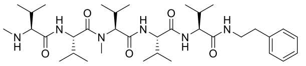
8a

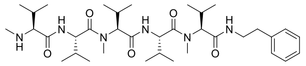
8b

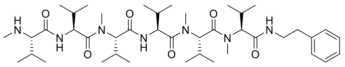
8d

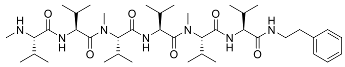
8c

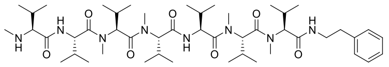
8e

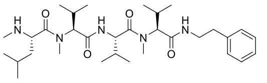
8f

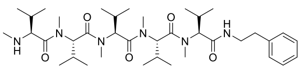
8g

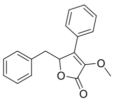
9

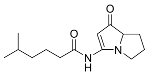
10

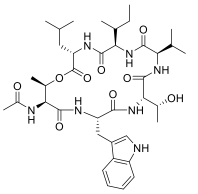
11

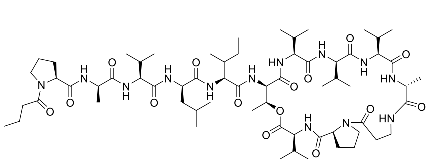
12a

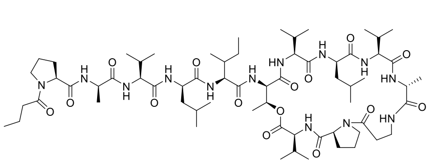
12b

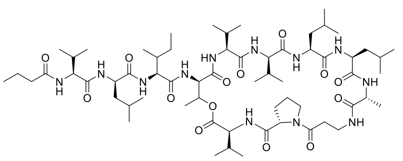
12c

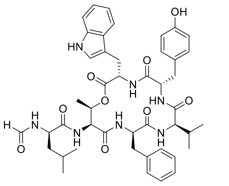
13

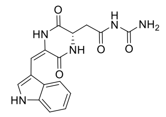
14

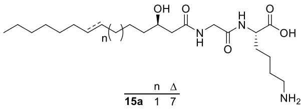

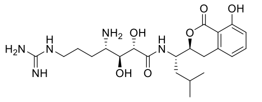
16a

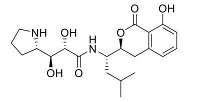
16b

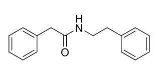
17a

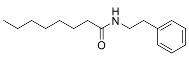
17b

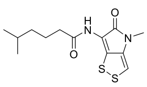
18a

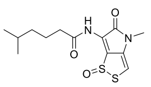
18b

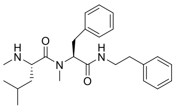
19

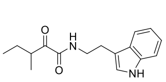
20

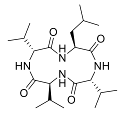
21

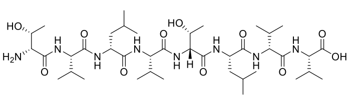

22

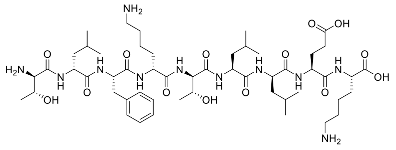

23

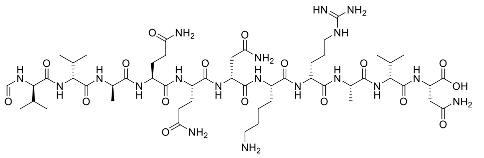

24a

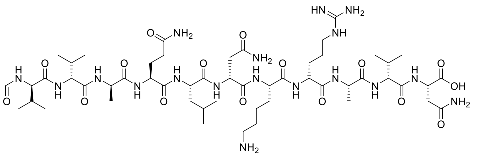

24b

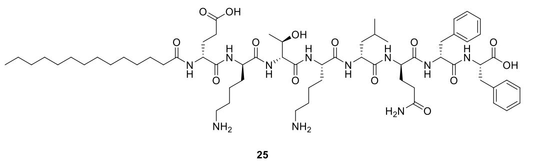

25

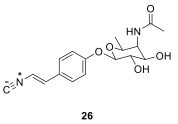

26

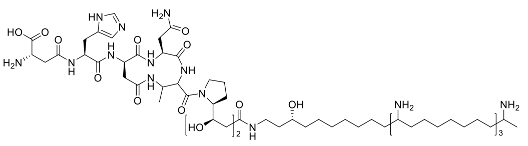

27

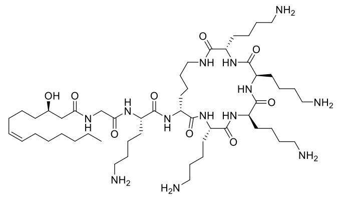

28

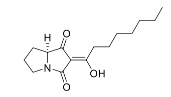

29a

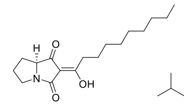

29b

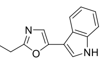

30a

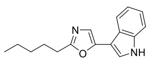

30b

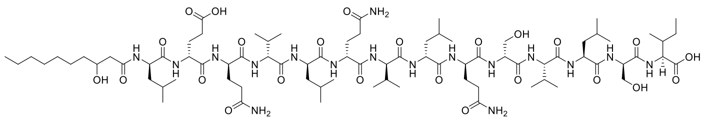

31a

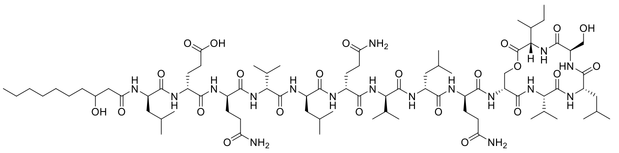

31b

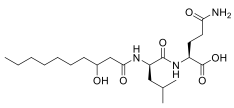

32a

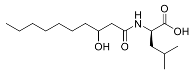

32b

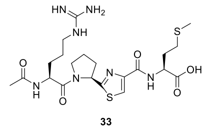

33

Supplementary Fig. 1. Structures of all NPs involved in the study.

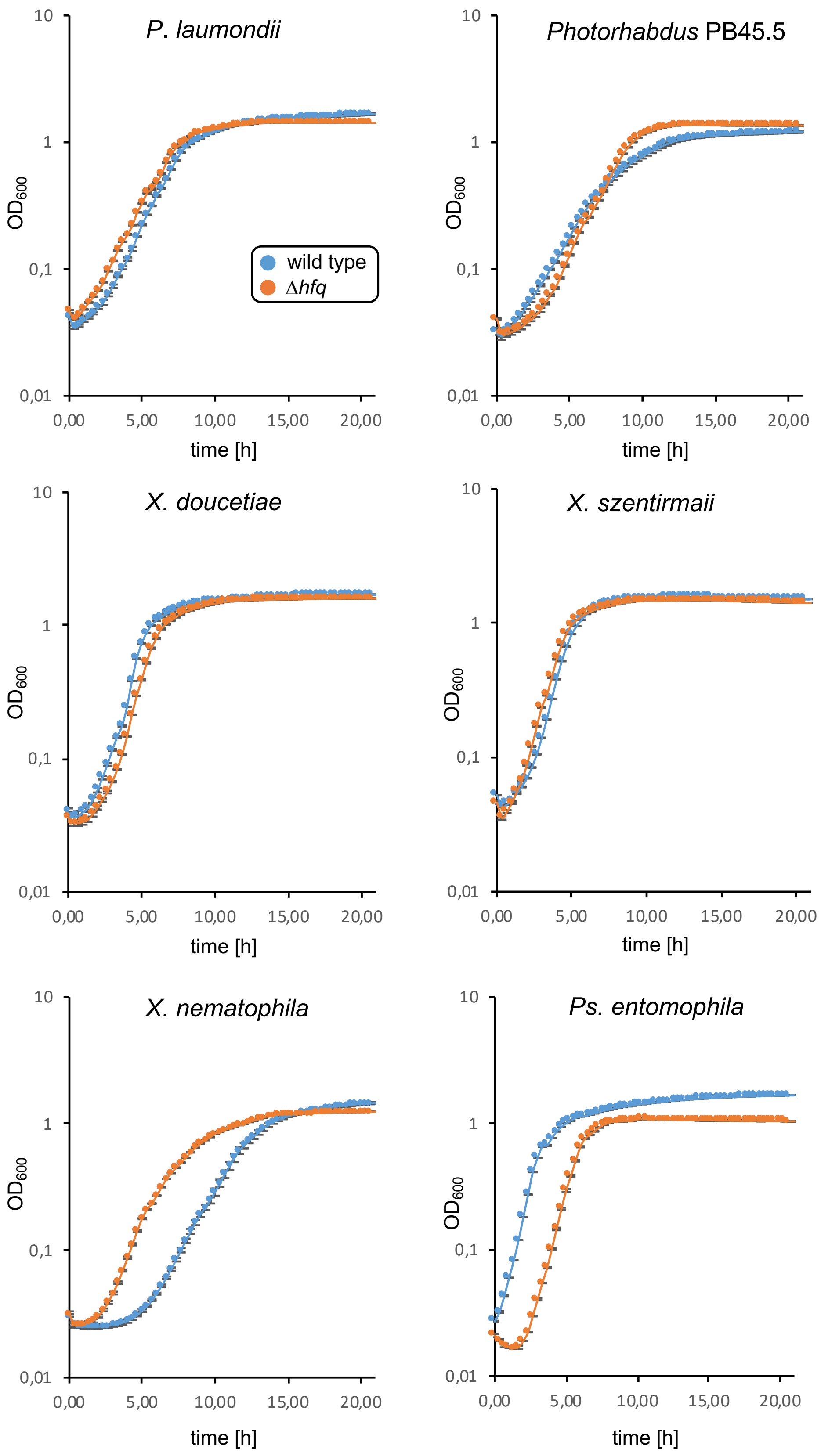

Supplementary Fig. 2. Comparison of growth of wild type (blue) and Δ*hfq* mutants (orange) used in this work. Data from triplicate experiments including standard deviations is shown.

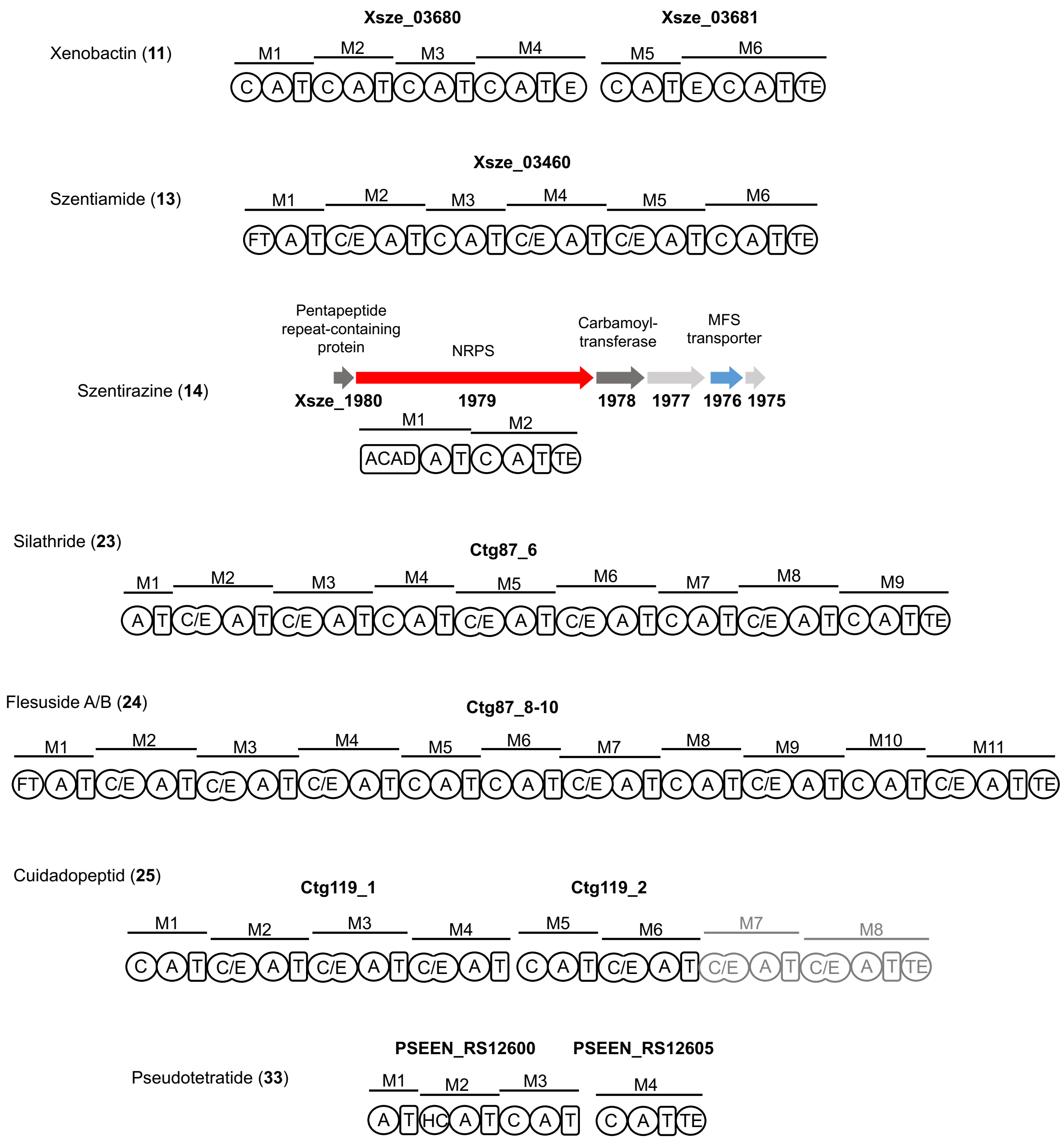

Supplementary Fig. 3. New NRPS and BGCs identified in this work. For **25** the C-terminus of the second NRPS was missing due to the BGC location at a contig end. The missing part (shown in grey) was assumed based on synthesis of all possible isomers (Supplementary Fig. 16c). Domain explanation: Condensation (C), dual condensation/epimerization (C/E), adenylation (A), thiolation (T), thioesterase (TE), formyltransferase (FT), heterocyclization (HC), acyl-CoA dehydrogenase (ACAD). Modules (M) are also shown.

a

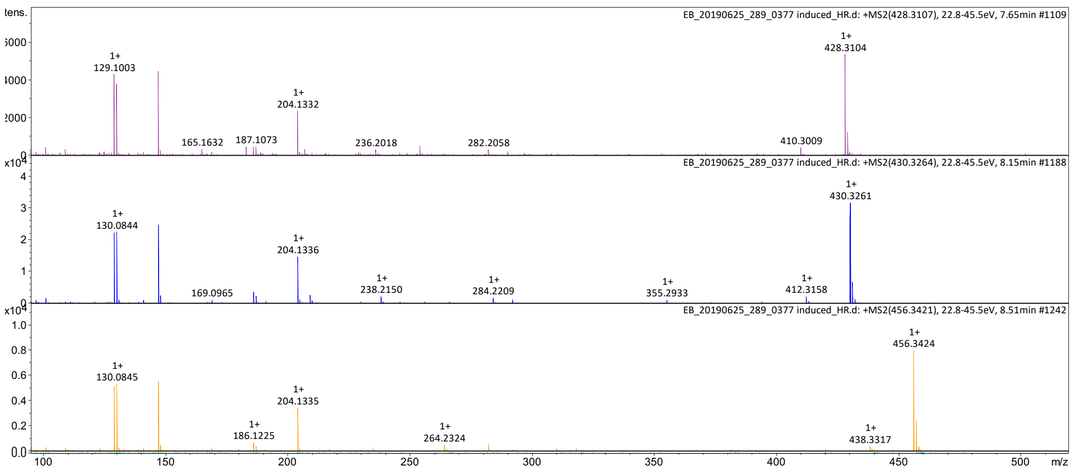

b

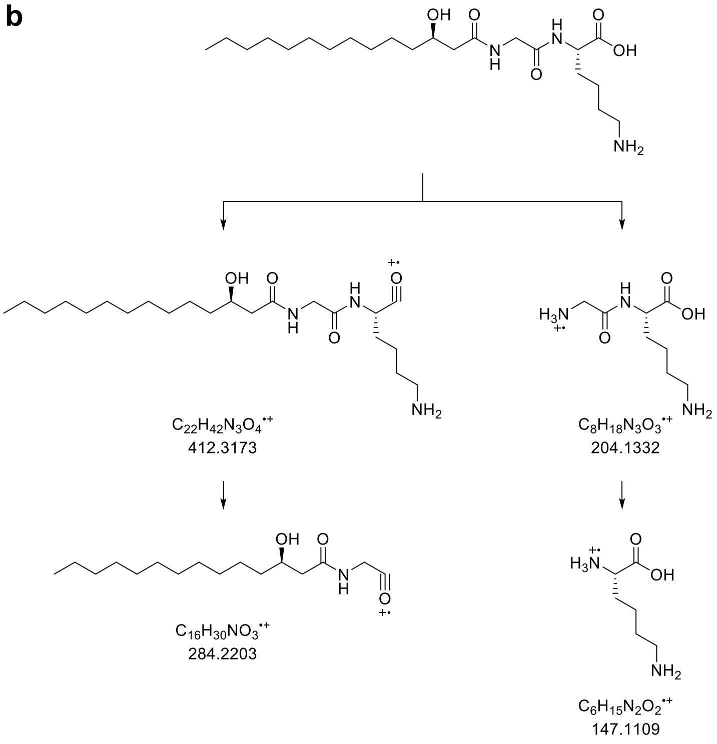

Supplementary Fig. 4. MS-MS analysis of **15a-c**. **a**, MS2-spectra of all identified derivatives. **b**, proposed fragmentation of **15b**.

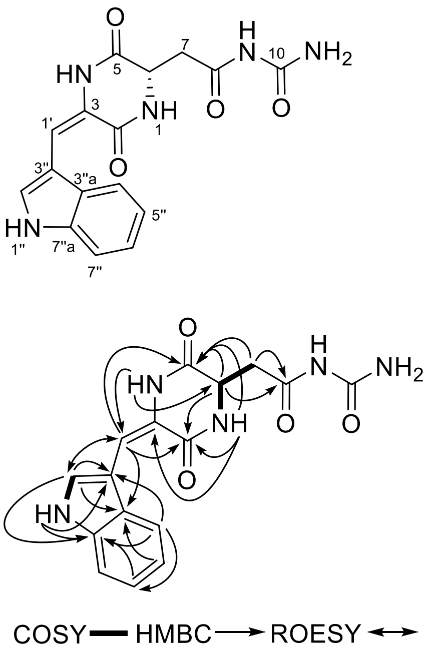

Supplementary Fig. 5. Structure and key 2D NMR correlations of **14**.

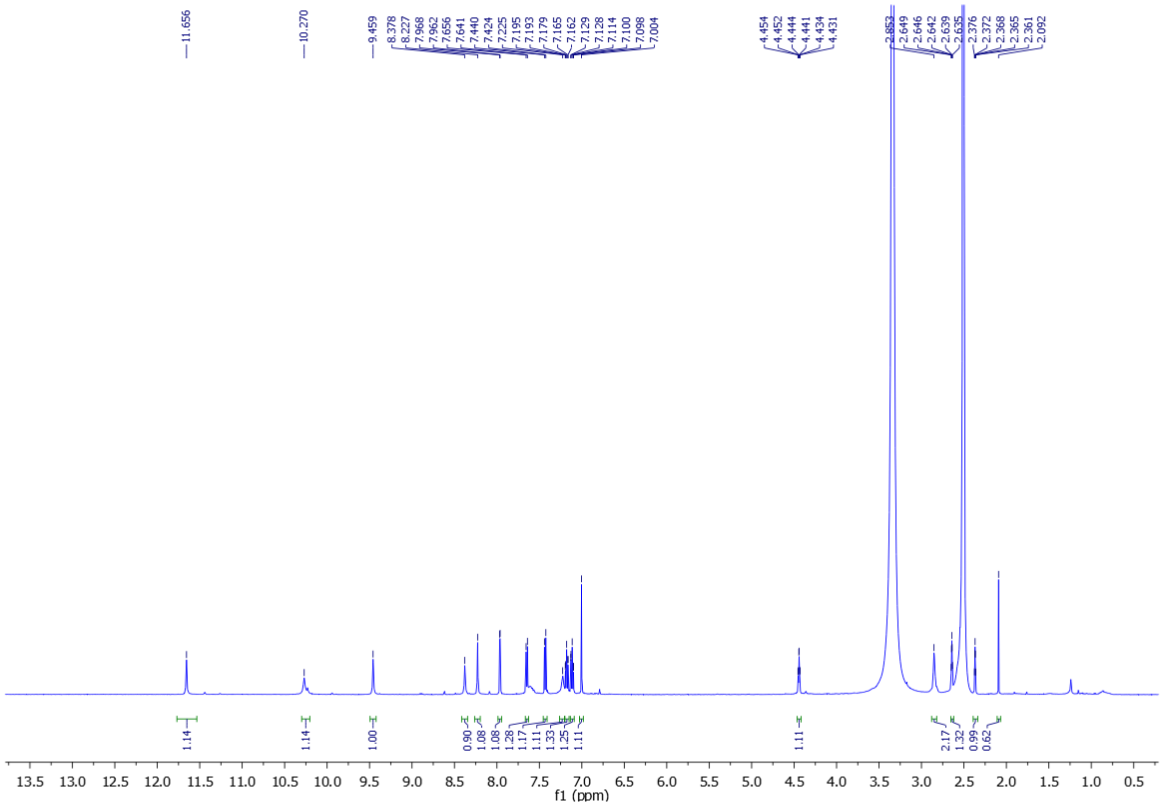

Supplementary Fig. 6. ^{1}H NMR spectrum of compound **14**.

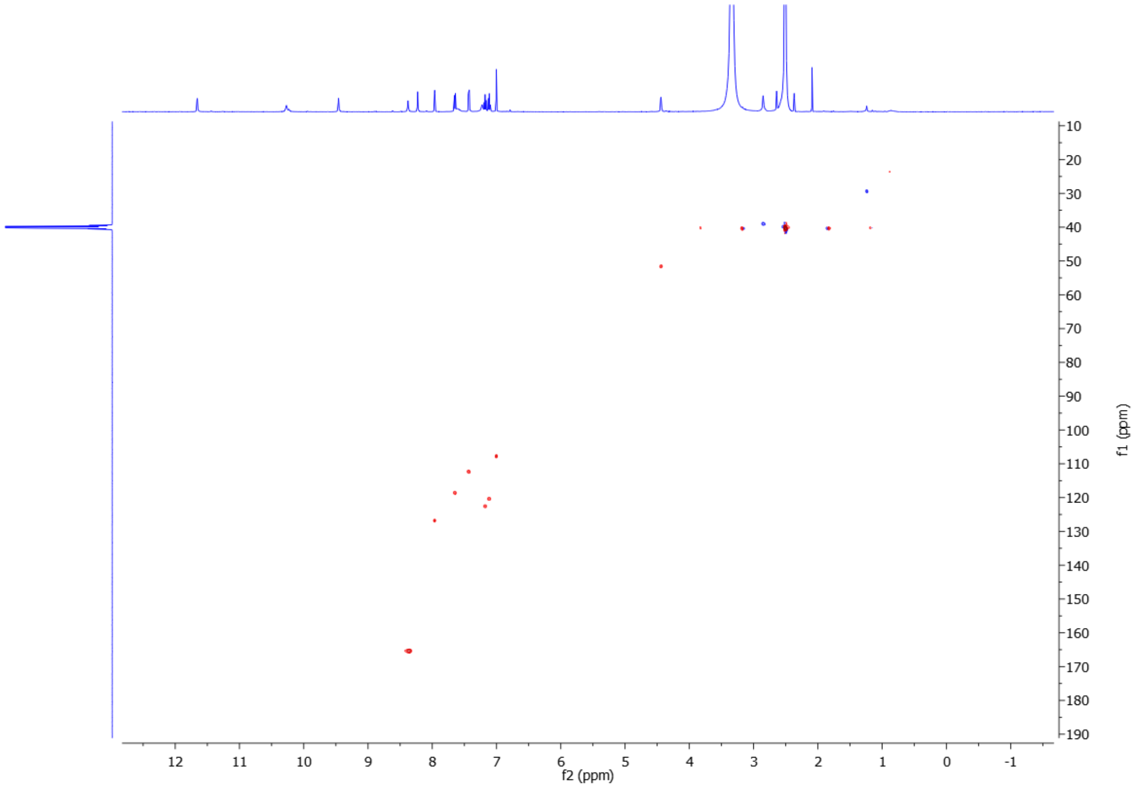

Supplementary Fig. 7. HSQC spectrum of compound **14**.

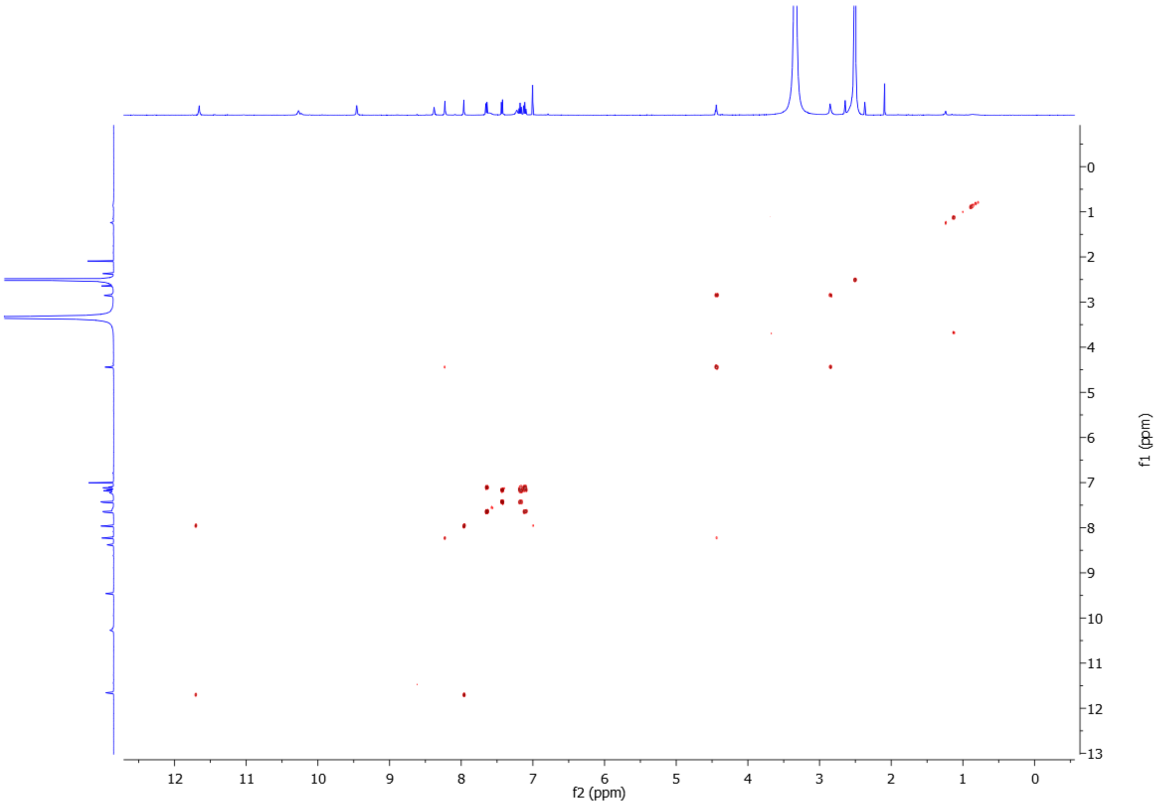

Supplementary Fig. 8. COSY spectrum of compound **14**.

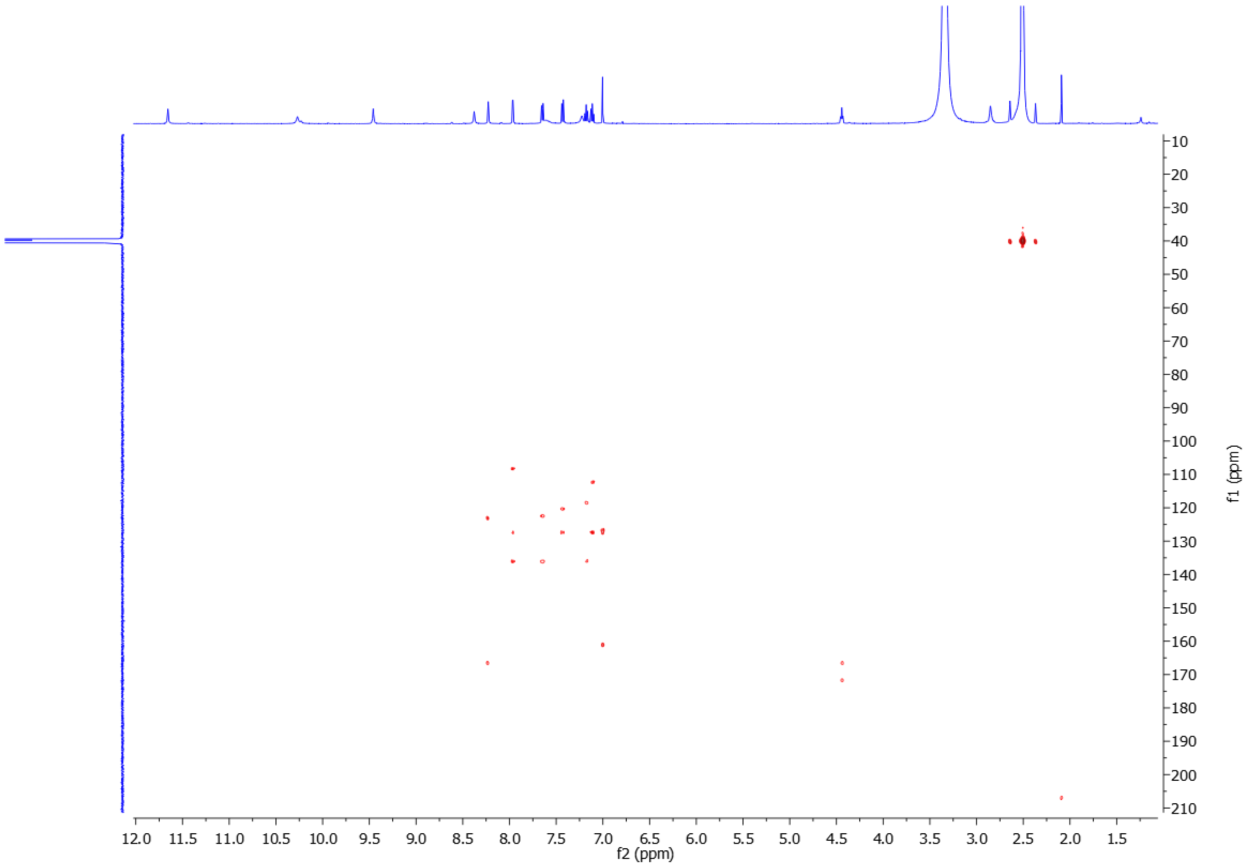

Supplementary Fig. 9. HMBC spectrum of compound **14**.

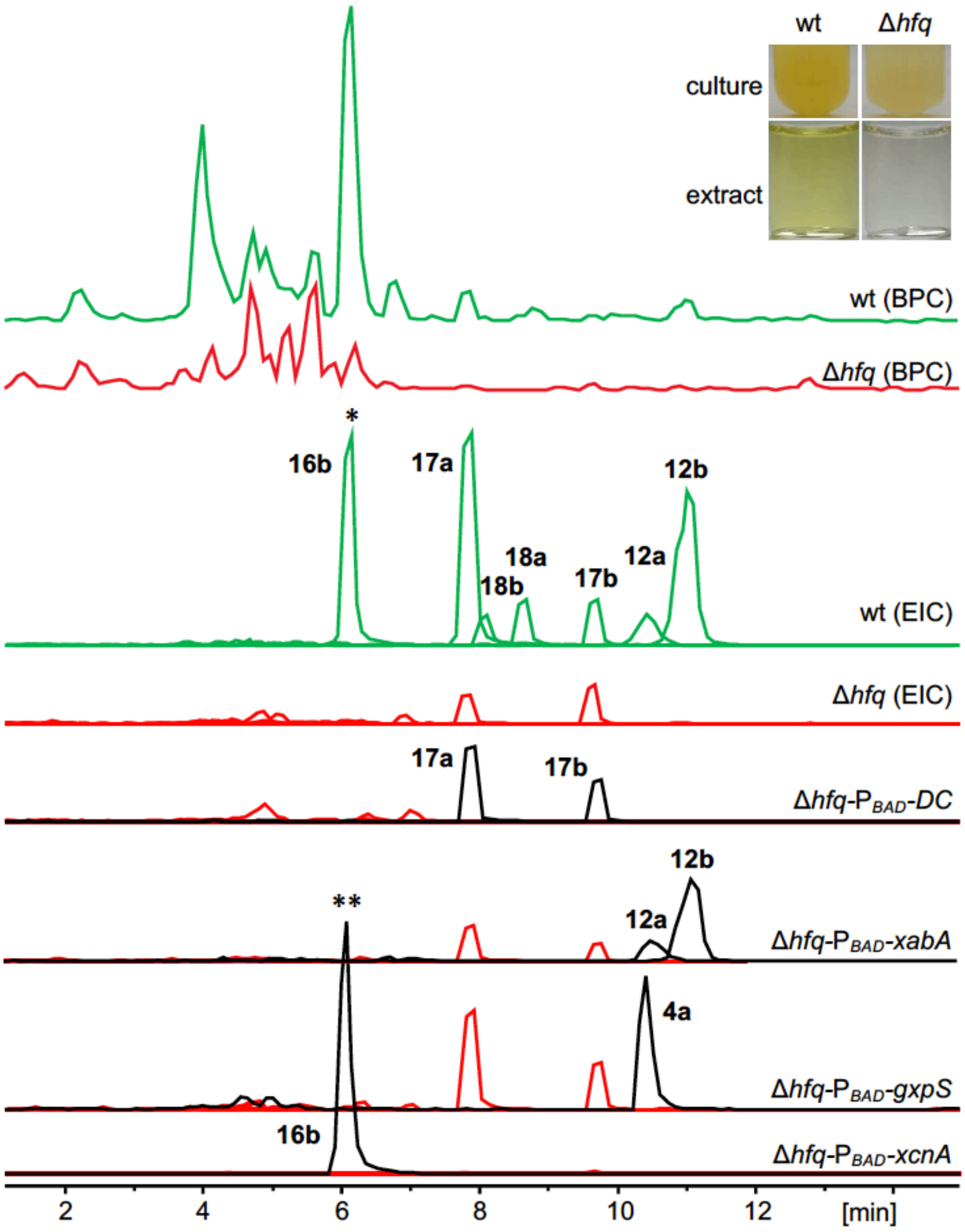

Supplementary Fig. 10. HPLC/MS data of wt and Δ*hfq* mutant of *X. doucetiae* and NPs detected after promoter exchange in the Δ*hfq* mutant. For better readability, overlaid Extracted Ion Chromatograms (EICs) of all NPs are shown clearly revealing the desired NPs upon promoter exchange. (* and **: 10- and 20-fold reduction in size).

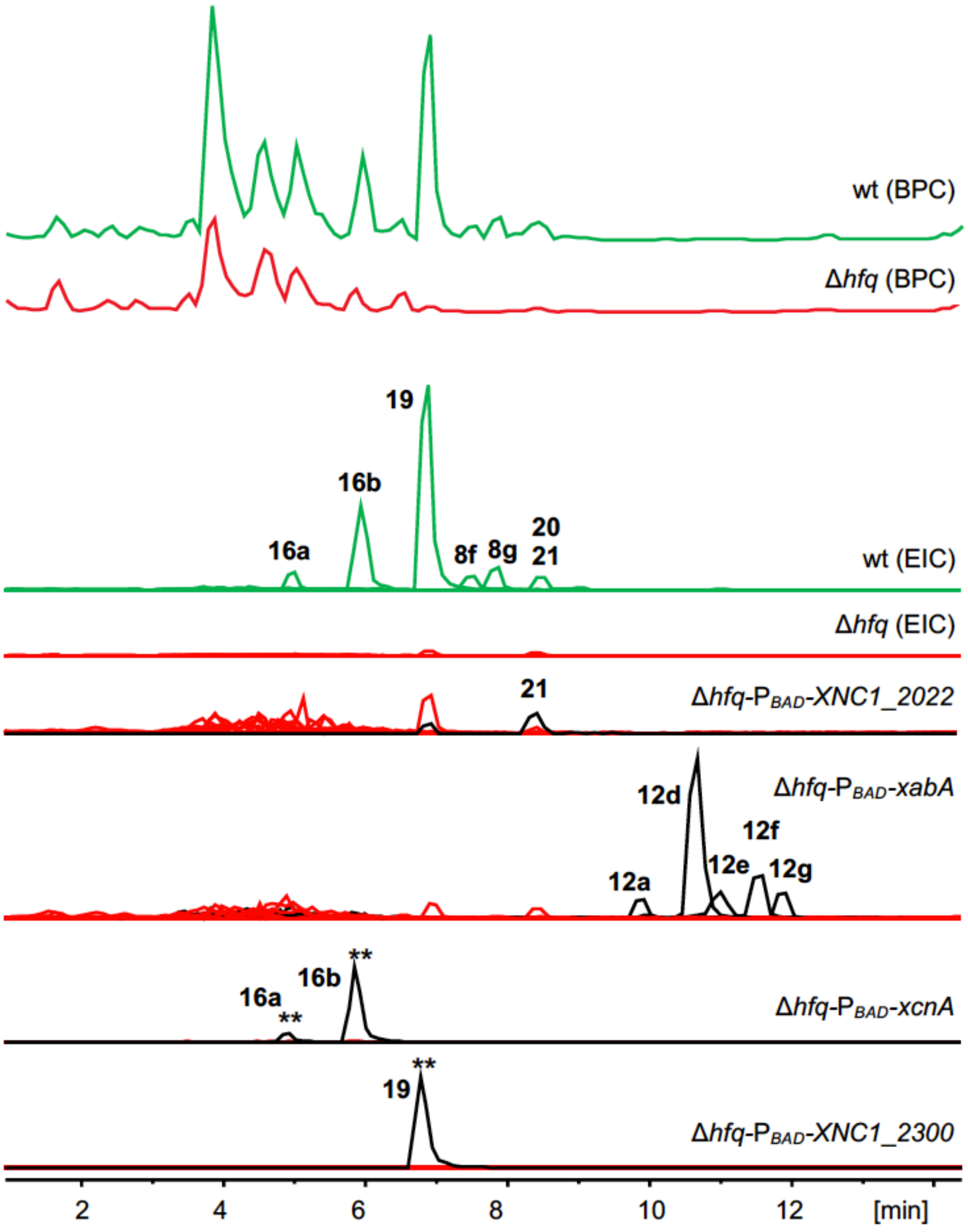

Supplementary Fig. 11. HPLC/MS data of wt and Δ*hfq* mutant of *X. nematophila* and NPs detected after promoter exchange in the Δ*hfq* mutant. For better readability, overlaid Extracted Ion Chromatograms (EICs) of all NPs are shown clearly revealing the desired NPs upon promoter exchange. (** 10-fold reduction in size).

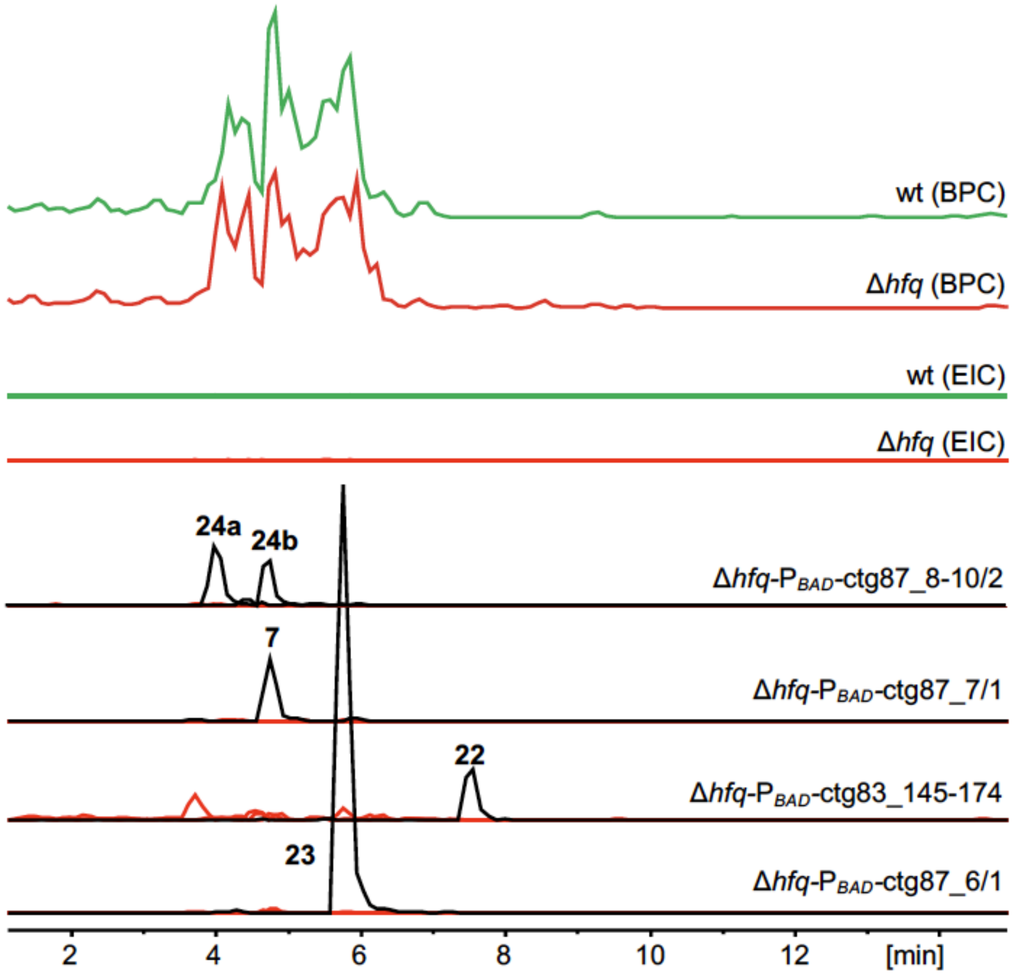

Supplementary Fig. 12. HPLC/MS data of wt and Δ*hfq* mutant of *Photorhabdus* PB45.5 and NPs detected after promoter exchange in the Δ*hfq* mutant. For better readability, overlaid Extracted Ion Chromatograms (EICs) of all NPs are shown clearly revealing the desired NPs upon promoter exchange.

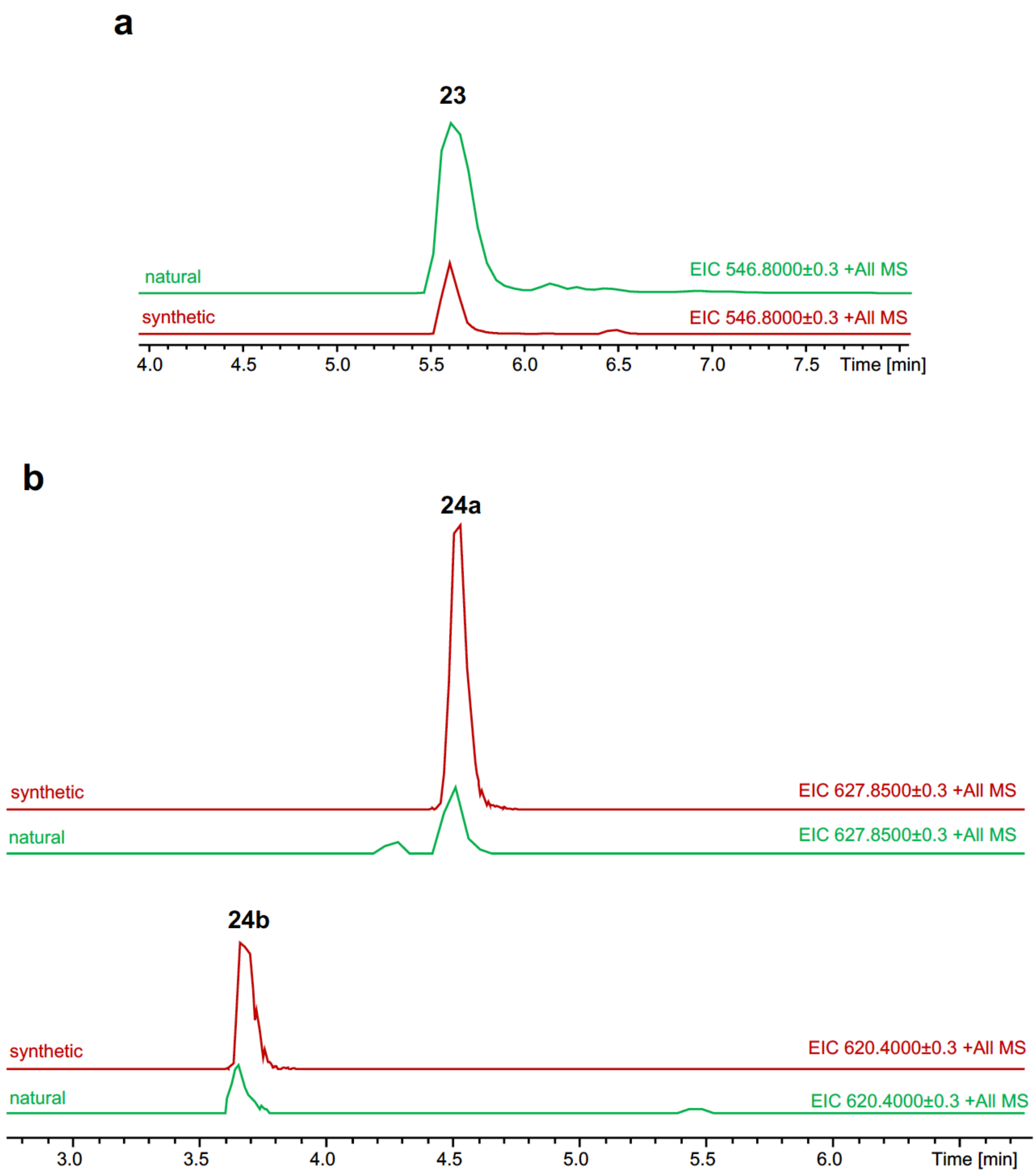

Supplementary Fig. 13. HPLC/MS analysis of synthetic (red) and natural (green) peptides **23** and **24** confirming their identical retention times and thus their absolute configurations.

a

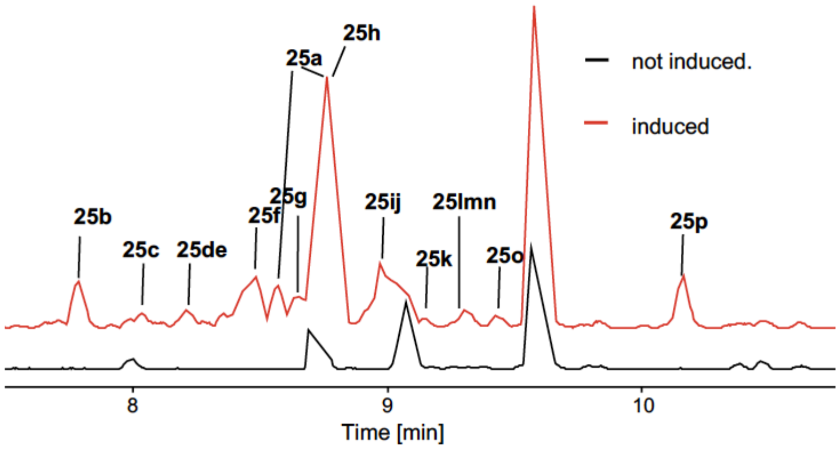

b

Derivative	**25b**	**25c**	**25d**	**25e**	**25f**
m/z $[M+2H]^{2+}$	414.3	421.3	611.4	635.4	618.4
RT (min)	7.9	8.06	8.3	8.3	8.6
Sum formula	$C_{41}H_{80}N_8O_9$	$C_{42}H_{82}N_8O_9$	$C_{62}H_{102}N_{12}O_{13}$	$C_{66}H_{102}N_{12}O_{13}$	$C_{63}H_{104}N_{12}O_{13}$
Derivative	**25a**	**25g**	**25h**	**25i**	**25j**
m/z $[M+2H]^{2+}$	625.4	631.4	608.4	615.4	632.4
RT (min)	8.8	8.67	8.8	8.98	8.98
Sum formula	$C_{64}H_{106}N_{12}O_{13}$	$C_{65}H_{106}N_{12}O_{13}$	$C_{61}H_{106}N_{12}O_{13}$	$C_{62}H_{110}N_{12}O_{13}$	$C_{65}H_{108}N_{12}O_{13}$
Derivative	**25k/25l**	**25m**	**25n**	**25o**	**25p**
m/z $[M+2H]^{2+}$	617.9	622.4	639.4	652.4	617.9
RT (min)	9.34	9.4	9.4	9.6	10.18
Sum formula	n.d.	$C_{63}H1_{12}N_{12}O_{13}$	$C_{66}H_{110}N_{12}O_{13}$	$C_{68}H_{112}N_{12}O_{13}$	n.d.

Supplementary Fig. 14. HPLC/MS (BPC) of *Xenorhabdus* KJ12.1 Δhfq_PBAD_02330 cultivated in in LB without (black) and with arabinose induction (red) (**a**) and data for cuidadopeptide derivatives (**25**) observed (main derivative **25a** highlighted).

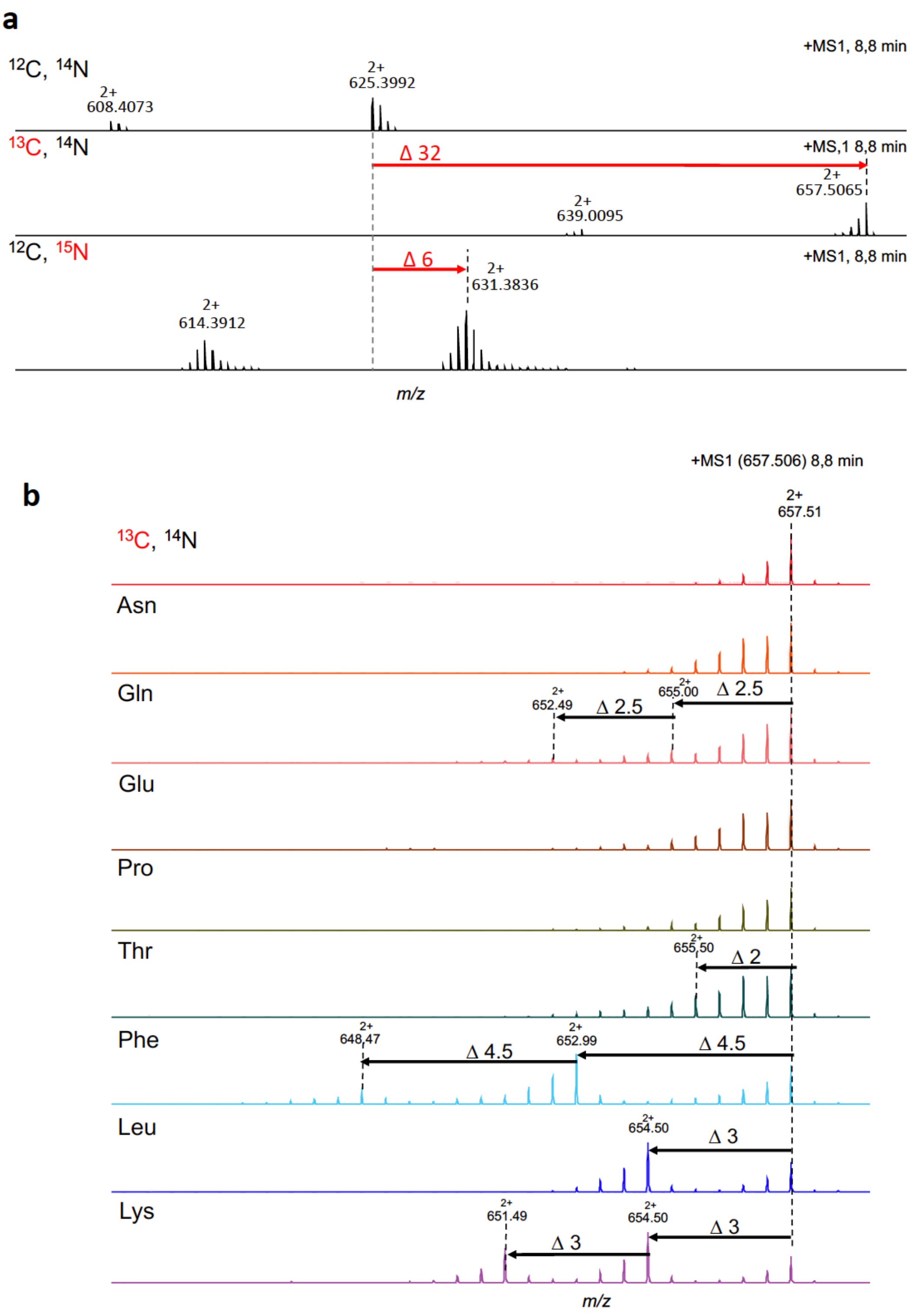

Supplementary Fig. 15. MS data from *Xenorhabdus* KJ12.1 Δhfq_PBAD_02330 producing **25a** grown in different media (fully labelled $^{12}C^{14}N$, $^{12}C^{15}N$, or $^{13}C^{14}N$) for the determination of carbon and nitrogen numbers (**a**) and amino acid building blocks determined from feeding non-labelled amino acids in cultures grown in ^{13}C medium (**b**).

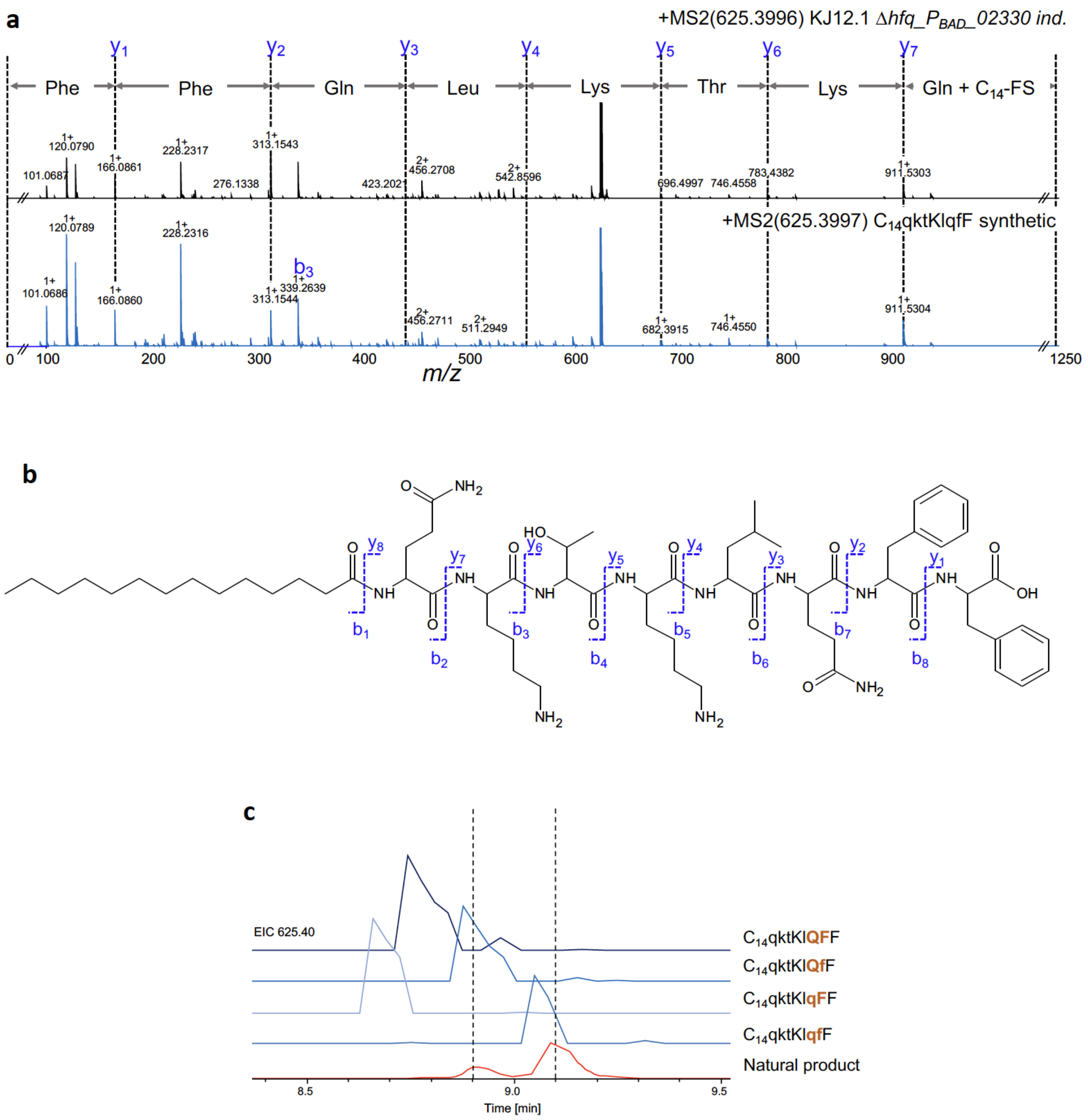

Supplementary Fig. 16. MS-MS Fragmentation of **25a** produced by *Xenorhabdus* KJ12.1 Δhfq_PBAD_02330 and compared to synthetic **25a** (**a**), proposed fragmentation pattern (**b**), and comparison of the retention time of synthetic and natural **25a** (**c**).

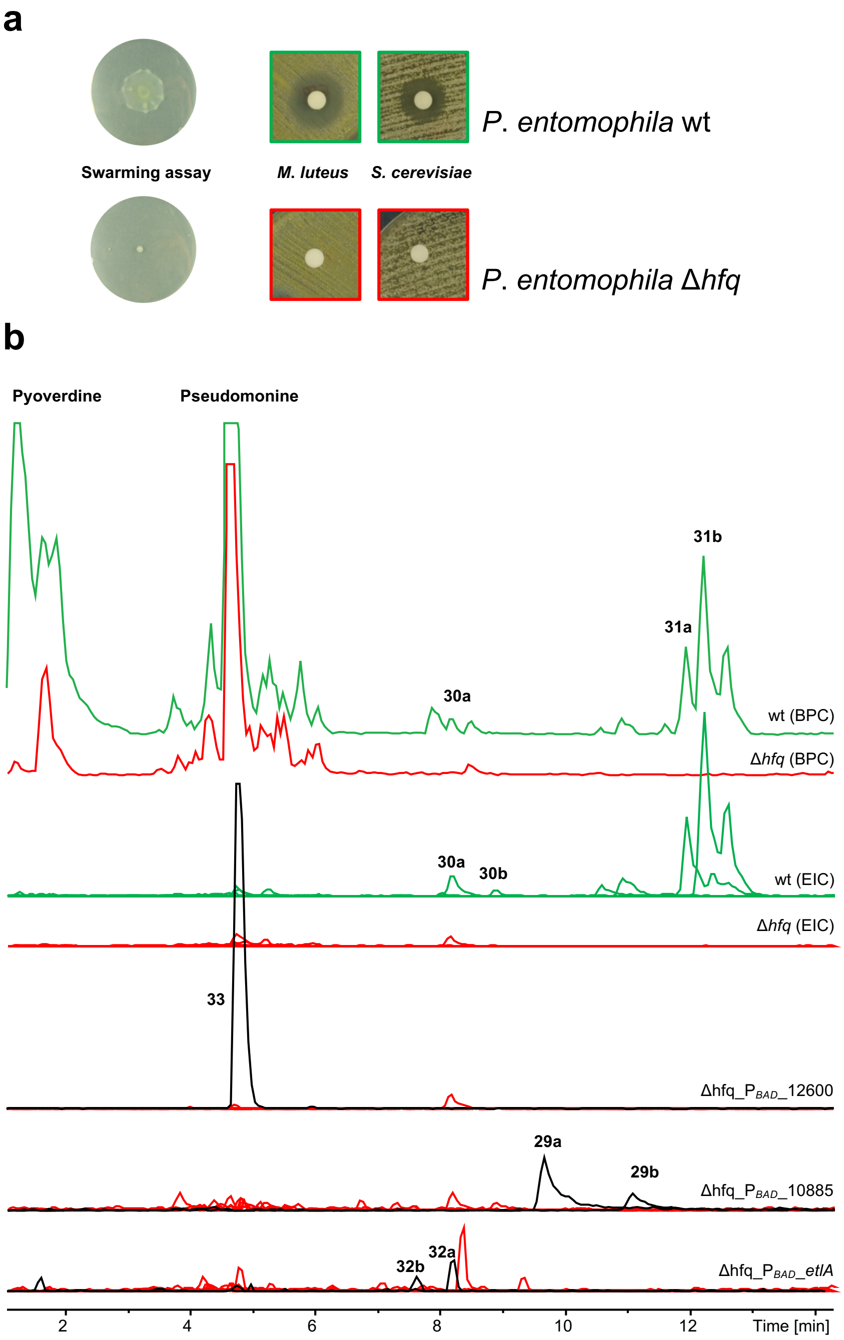

Supplementary Fig. 17. HPLC/MS data of wt and Δ*hfq* mutant of *Pseudomonas entomophila* and NPs detected after promoter exchange in the Δ*hfq* mutant. For better readability, overlaid Extracted Ion Chromatograms (EICs) of all NPs are shown clearly revealing the desired NPs upon promoter exchange. In the Δ*hfq* mutant the two siderophores pyoverdine and pseudomonine were still detected but were left out in the EIC representation for better readability. EIC traces for **29** and **32** were increased 10-fold.

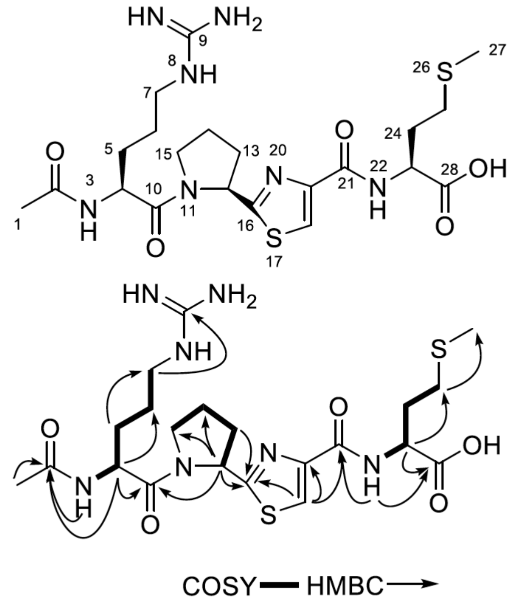

Supplementary Fig. 18. Structure and key 2D NMR correlations of **33**.

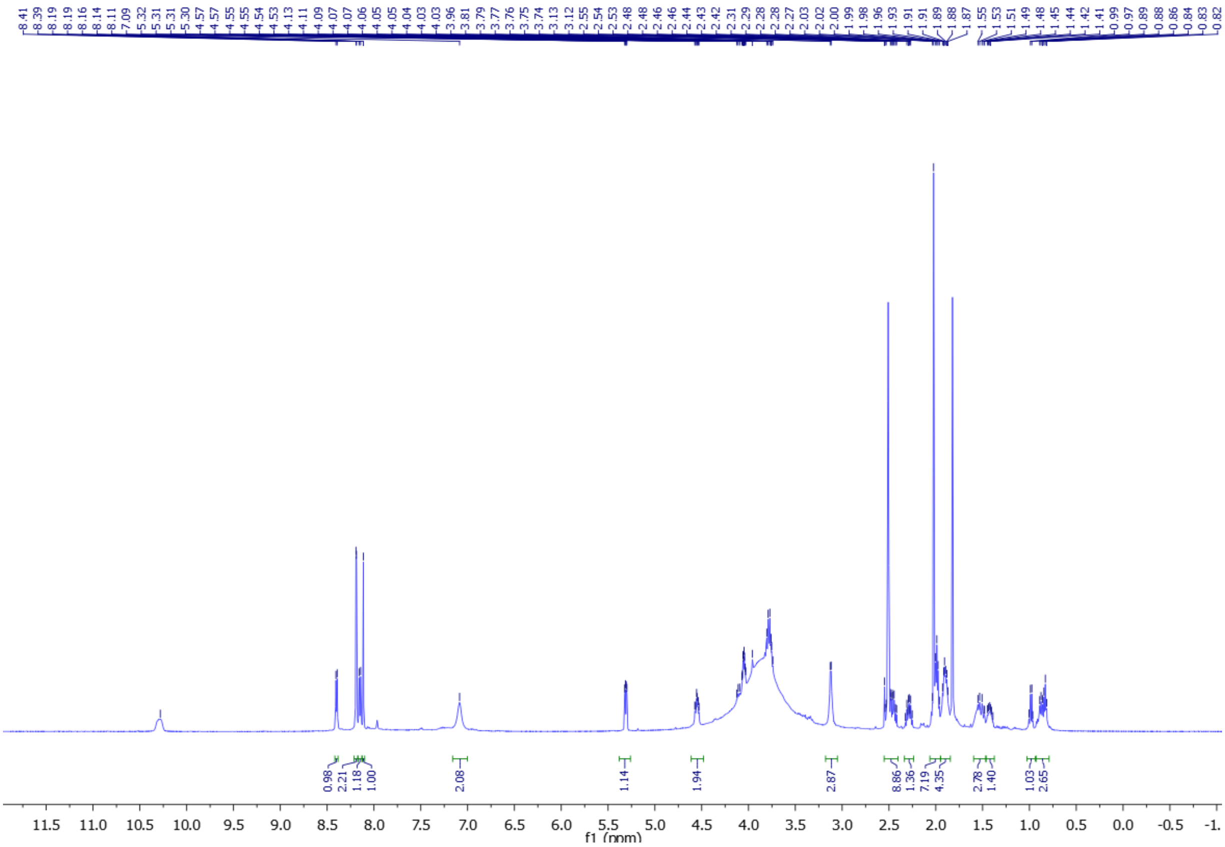

Supplementary Fig. 19. ^{1}H NMR spectrum of compound **33**.

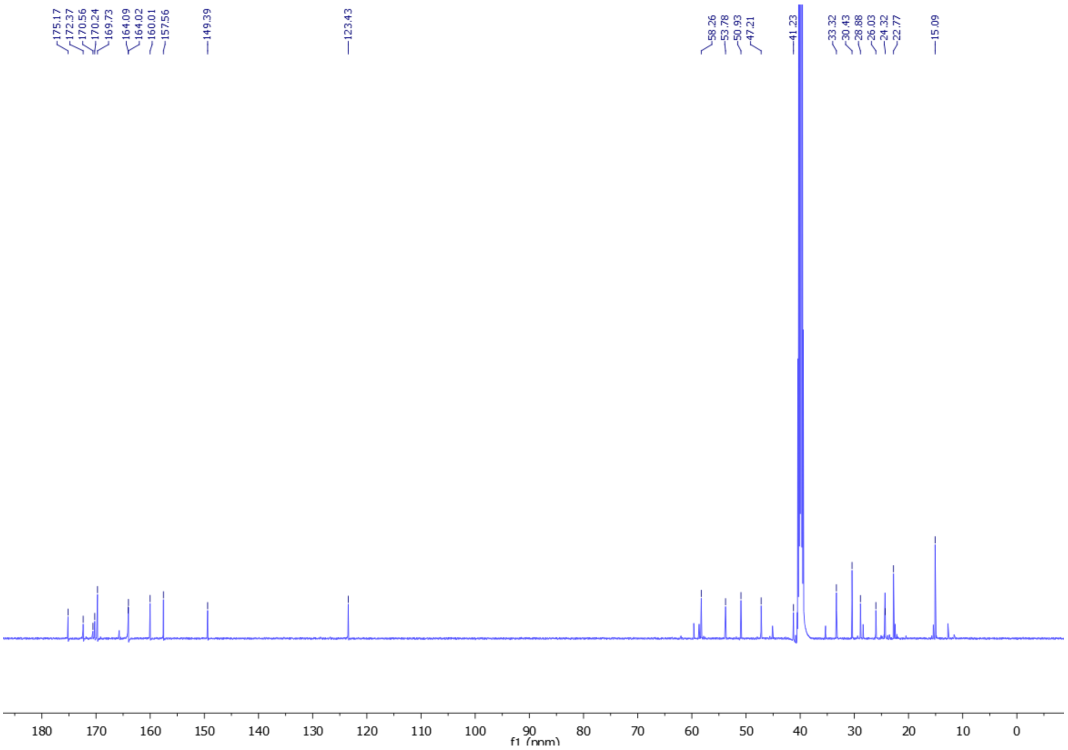

Supplementary Fig. 20. ^{13}C NMR spectrum of compound **33**.

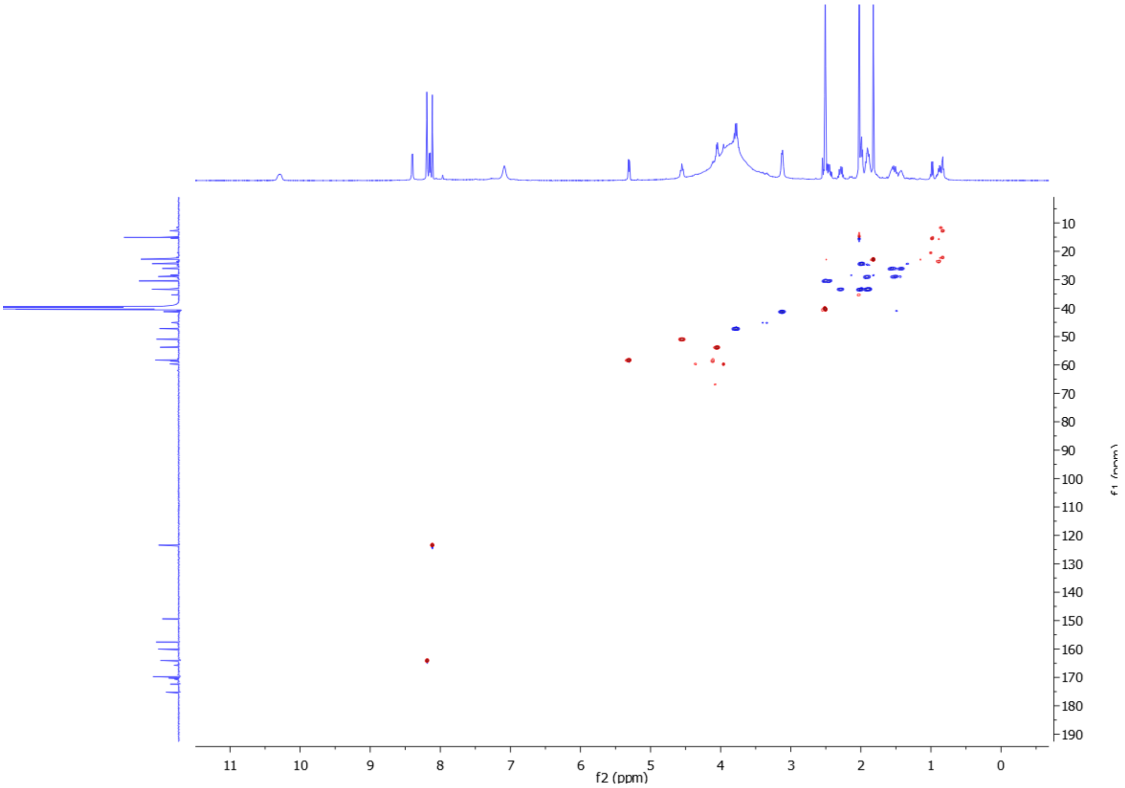

Supplementary Fig. 21. HSQC spectrum of compound **33**.

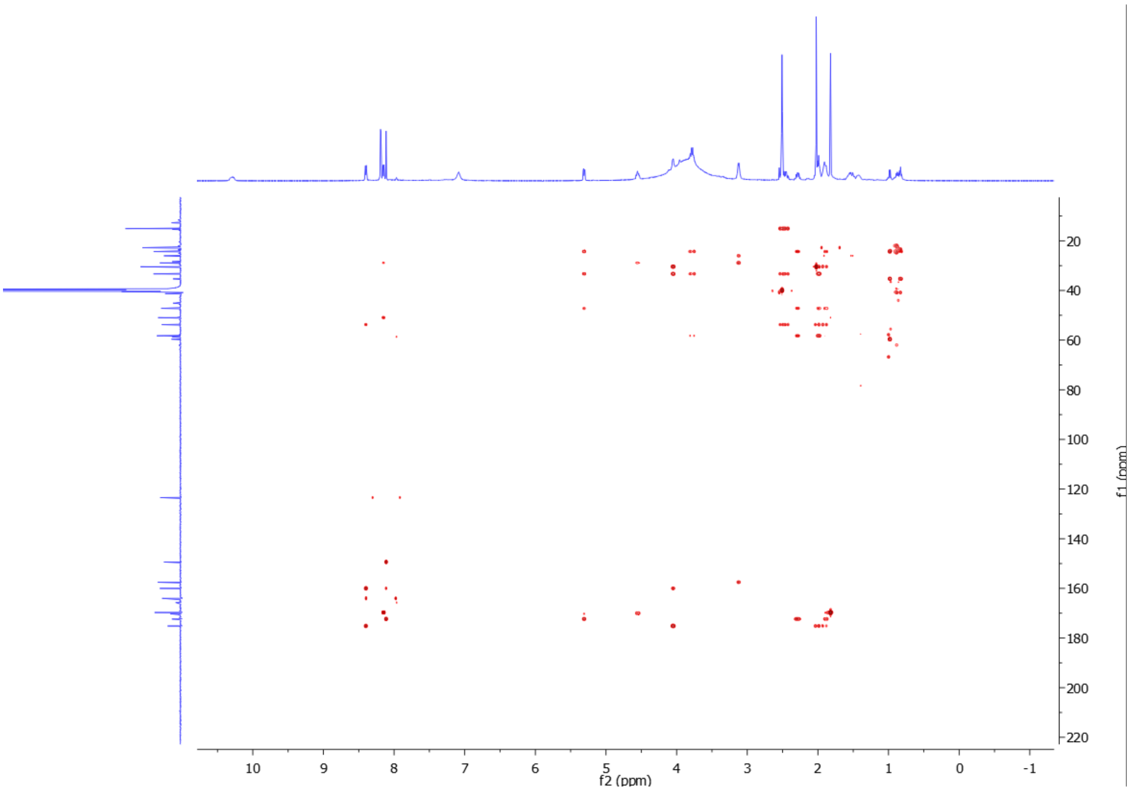

Supplementary Fig. 22. HMBC spectrum of compound **33**.

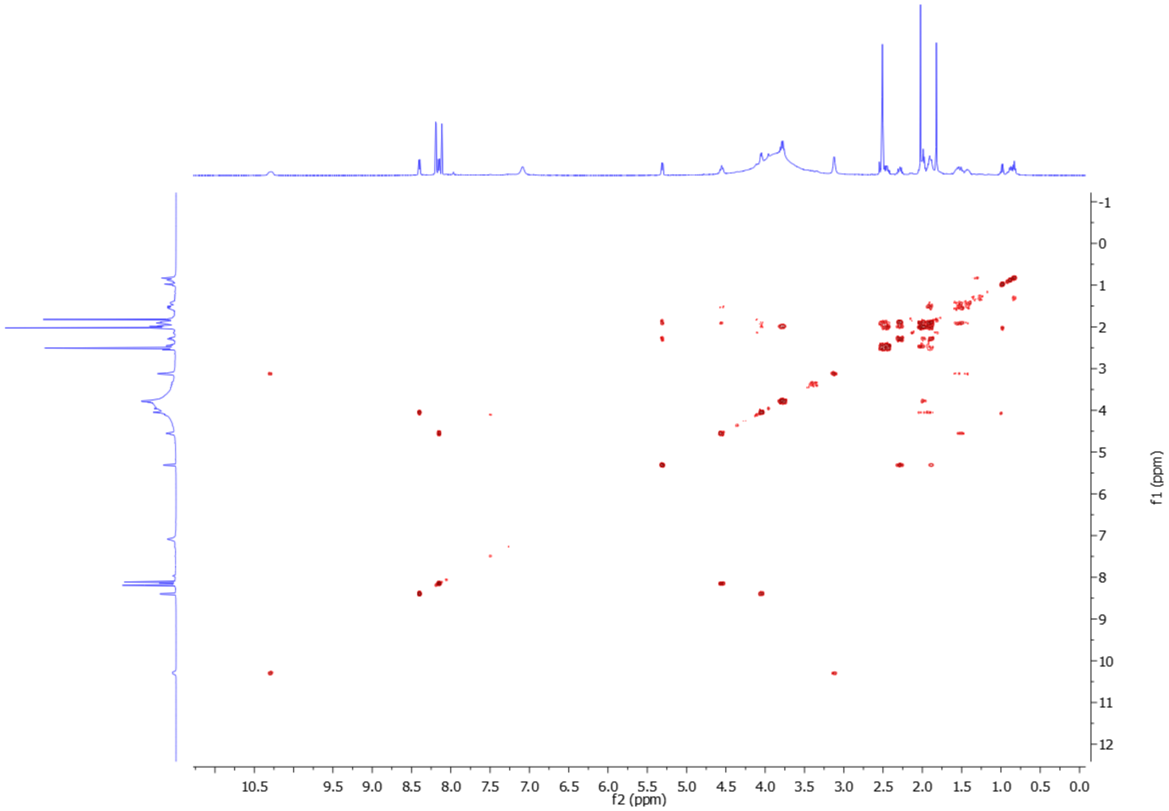

Supplementary Fig. 23. COSY spectrum of compound **33**.

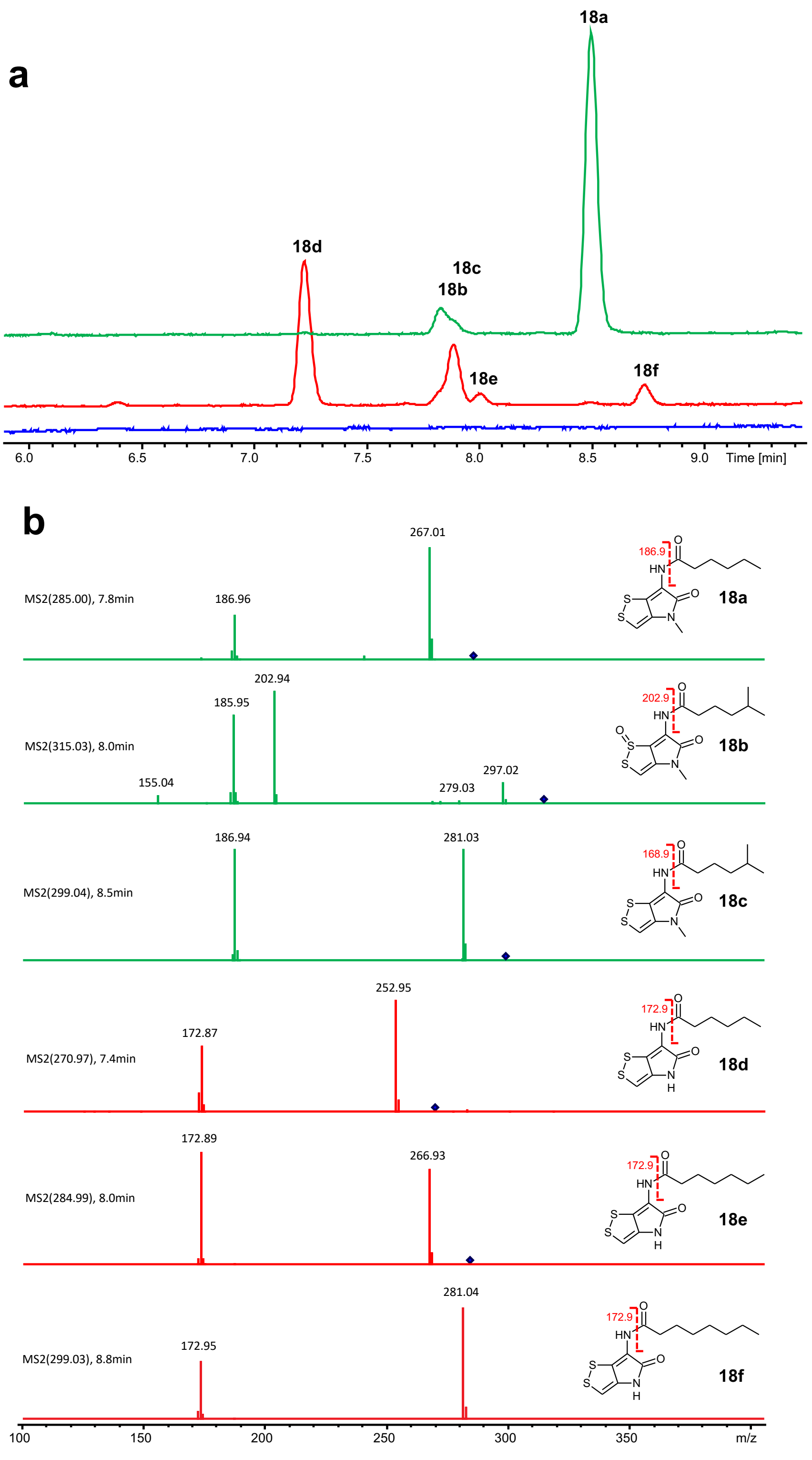

Supplementary Fig. 24. a, HPLC/UV analysis of xenorhabdins derived from *X. doucetiae* wt (green)(**18a-c**), induced *X. doucetiae* Δ*hfq-xrdA* mutant (red)(**18d-f**) and non-induced *X. doucetiae* Δ*hfq-xrdA* mutant (blue). **b**, MS-MS analysis of the identified derivatives indicating characteristic fragments.

References

[1] N. J. Tobias, A. K. Heinrich, H. Eresmann, P. R. Wright, N. Neubacher, R. Backofen, H. B. Bode, *Environ Microbiol* **2017**, *19*, 119–129.

[2] C. Fu, W. P. Donovan, O. Shikapwashya-Hasser, X. Ye, R. H. Cole, *PLoS ONE* **2014**, *9*, e115318–20.

[3] L. R. Hmelo, B. R. Borlee, H. Almblad, M. E. Love, T. E. Randall, B. S. Tseng, C. Lin, Y. Irie, K. M. Storek, J. J. Yang, et al., *Nat Protoc* **2015**, *10*, 1820–1841.

[4] E. Bode, A. O. Brachmann, C. Kegler, R. Simsek, C. Dauth, Q. Zhou, M. Kaiser, P. Klemmt, H. B. Bode, *ChemBioChem* **2015**, *16*, 1115–1119.

[5] S. Thoma, M. Schobert, *FEMS Microbiol Lett* **2009**, *294*, 127–132.

[6] N. J. Tobias, H. Wolff, B. Djahanschiri, F. Grundmann, M. Kronenwerth, Y.-M. Shi, S. Simonyi, P. Grün, D. Shapiro-Ilan, S. J. Pidot, et al., *Nature Microbiology* **2017**, *2*, 1676–1685.

[7] D. Sørensen, T. O. Larsen, C. Christophersen, P. H. Nielsen, U. Anthoni, *Phytochemistry* **1999**, *51*, 1181–1183.

[8] S. W. Fuchs, A. Proschak, T. W. Jaskolla, M. Karas, H. B. Bode, *Org. Biomol. Chem.* **2011**, *9*, 3130–3132.

[9] C. J. Balibar, F. H. Vaillancourt, C. T. Walsh, *Chem. Biol.* **2005**, *12*, 1189–1200.

[10] K. Blin, S. Shaw, K. Steinke, R. Villebro, N. Ziemert, S. Y. Lee, M. H. Medema, T. Weber, *Nucleic Acids Res.* **2019**, *47*, W81–W87.

[11] J. Leach, B. R. Lang, O. Y. Microbiology, 1982, *J. Gen. Microbiol.* **1982**, *128*, 1719–1729.

[12] S. Hazir, D. I. Shapiro-Ilan, C. H. Bock, C. Hazir, L. G. Leite, M. W. Hotchkiss, *European Journal of Plant Pathology* **2016**, *146*, 1–13.

[13] X. L. Fang, Z. Z. Li, Y. H. Wang, X. Zhang, *J Appl Microbiol* **2011**, *111*, 145–154.

[14] E. Matuschek, D. F. J. Brown, G. Kahlmeter, *Clinical Microbiology and Infection* **2014**, *20*, O255–O266.

[15] C. L. Kurz, S. Chauvet, E. Andrès, M. Aurouze, I. Vallet, G. P. F. Michel, M. Uh, J. Celli, A. Filloux, S. De Bentzmann, et al., *EMBO J.* **2003**, *22*, 1451–1460.

[16] S. Brenner, *Genetics* **1974**, *77*, 71–94.

[17] I. Cakmak, H. Baspinar, N. MADANLAR, *Turk J Agric For* **2005**, *29*, 259–265.

[18] I. Cakmak, A. Janssen, M. W. Sabelis, H. Baspinar, *Biological Control* **2009**, *50*, 60–65.

[19] D. I. Quiros, D. A. Emmen, E. Dominguez, M. V. Heller, P. D. Coley, T. A. Kursar, *International Journal of Pest Management* **2006**, *52*, 333–342.

[20] E. W. Ades, F. J. Candal, R. A. Swerlick, V. G. George, S. Summers, D. C. Bosse, T. J. Lawley, *Journal of Investigative Dermatology* **1992**, *99*, 683–690.

[21] P. Griess, *Berichte der deutschen chemischen Gesellschaft* **1879**, *12*, 426–428.

[22] A. O. Brachmann, S. A. Joyce, H. Jenke-Kodama, G. Schwär, D. J. Clarke, H. B. Bode, *ChemBioChem* **2007**, *8*, 1721–1728.

[23] J. Li, G. Chen, H. Wu, J. M. Webster, *Appl Environ Microbiol* **1995**, *61*, 4329–4333.

[24] S. A. Joyce, A. O. Brachmann, I. Glazer, L. Lango, G. Schwär, D. J. Clarke, H. B. Bode, *Angew. Chem. Int. Ed. Engl.* **2008**, *47*, 1942–1945.

[25] F. I. Nollmann, A. K. Heinrich, A. O. Brachmann, C. Morisseau, K. Mukherjee, Á. M. Casanova-Torres, F. Strobl, D. Kleinhans, S. Kinski, K. Schultz, et al., *ChemBioChem* **2015**, *16*, 766–771.

[26] H. B. Bode, D. Reimer, S. W. Fuchs, F. Kirchner, C. Dauth, C. Kegler, W. Lorenzen, A. O. Brachmann, P. Grün, *Chem. Eur. J.* **2012**, *18*, 2342–2348.
[27] F. I. Nollmann, C. Dauth, G. Mulley, C. Kegler, M. Kaiser, N. R. Waterfield, H. B. Bode, *ChemBioChem* **2015**, *16*, 205–208.
[28] A. O. Brachmann, S. Brameyer, D. Kresovic, I. Hitkova, Y. Kopp, C. Manske, K. Schubert, H. B. Bode, R. Heermann, *Nat Chem Biol* **2013**, *9*, 573–578.
[29] A. Dudnik, L. Bigler, R. Dudler, *Microbiol Res* **2013**, *168*, 73–76.
[30] C. M. Theodore, J. B. King, J. You, R. H. Cichewicz, *J. Nat. Prod.* **2012**, *75*, 2007–2011.
[31] G. Wang, Z. Zhao, J. Ke, Y. Engel, Y.-M. Shi, *Nature Microbiology* **n.d.**
[32] D. Reimer, K. N. Cowles, A. Proschak, F. I. Nollmann, A. J. Dowling, M. Kaiser, R. F. Constant, H. Goodrich-Blair, H. B. Bode, *ChemBioChem* **2013**, *14*, 1991–1997.
[33] X. Cai, S. Nowak, F. Wesche, I. Bischoff, M. Kaiser, R. Fürst, H. B. Bode, *Nat Chem* **2017**, *9*, 379–386.
[34] A. O. Brachmann, S. Forst, G. M. Furgani, A. Fodor, H. B. Bode, *J. Nat. Prod.* **2006**, *69*, 1830–1832.
[35] O. Schimming, V. L. Challinor, N. J. Tobias, H. Adihou, P. Grün, L. Pöschel, C. Richter, H. Schwalbe, H. B. Bode, *Angewandte Chemie* **2015**, *127*, 12893–12896.
[36] F. Grundmann, M. Kaiser, M. Kurz, M. Schiell, A. Batzer, H. B. Bode, *RSC Adv.* **2013**, *3*, 22072–22077.
[37] Q. Zhou, F. Grundmann, M. Kaiser, M. Schiell, S. Gaudriault, A. Batzer, M. Kurz, H. B. Bode, *Chem. Eur. J.* **2013**, *19*, 16772–16779.
[38] B. Ohlendorf, S. Simon, J. Wiese, J. F. Imhoff, *Nat Prod Commun* **2011**, *6*, 1247–1250.
[39] F. I. Nollmann, A. Dowling, M. Kaiser, K. Deckmann, S. Grösch, R. ffrench-Constant, H. B. Bode, *Beilstein J. Org. Chem.* **2012**, *8*, 528–533.
[40] D. Ji, Y. Yi, G.-H. Kang, Y.-H. Choi, P. Kim, N.-I. Baek, Y. Kim, *FEMS Microbiol Lett* **2004**, *239*, 241–248.
[41] D. Reimer, E. Luxenburger, A. O. Brachmann, H. B. Bode, *ChemBioChem* **2009**, *10*, 1997–2001.
[42] E. Bode, Y. He, T. D. Vo, R. Schultz, M. Kaiser, H. B. Bode, *Environ Microbiol* **2017**, *19*, 4564–4575.
[43] B. V. McInerney, R. P. Gregson, M. J. Lacey, R. J. Akhurst, G. R. Lyons, S. H. Rhodes, D. R. Smith, L. M. Engelhardt, A. H. White, *J. Nat. Prod.* **1991**, *54*, 774–784.
[44] D. Reimer, F. I. Nollmann, K. Schultz, M. Kaiser, H. B. Bode, *J. Nat. Prod.* **2014**, *77*, 1976–1980.
[45] X. Cai, V. L. Challinor, L. Zhao, D. Reimer, H. Adihou, P. Grün, M. Kaiser, H. B. Bode, *Org. Lett.* **2017**, *19*, 806–809.
[46] C. Kegler, F. I. Nollmann, T. Ahrendt, F. Fleischhacker, E. Bode, H. B. Bode, *ChemBioChem* **2014**, *15*, 826–828.
[47] H. B. Bode, A. O. Brachmann, K. B. Jadhav, L. Seyfarth, C. Dauth, S. W. Fuchs, M. Kaiser, N. R. Waterfield, H. Sack, S. H. Heinemann, et al., *Angew. Chem. Int. Ed. Engl.* **2015**, *54*, 10352–10355.
[48] J. M. Crawford, C. Portmann, X. Zhang, M. B. J. Roeffaers, J. Clardy, *Proc. Natl. Acad. Sci. U.S.A.* **2012**, *109*, 10821–10826.
[49] S. W. Fuchs, F. Grundmann, M. Kurz, M. Kaiser, H. B. Bode, *ChemBioChem* **2014**, *15*, 512–516.

[50] M. Klapper, S. Götze, R. Barnett, K. Willing, P. Stallforth, *Angewandte Chemie* **2016**, *128*, 9090–9093.

[51] F. Grundmann, V. Dill, A. Dowling, A. Thanwisai, E. Bode, N. Chantratita, R. ffrench-Constant, H. B. Bode, *Beilstein J. Org. Chem.* **2012**, *8*, 749–752.

[52] A. Broberg, J. Bjerketorp, P. Andersson, C. Sahlberg, J. Levenfors, *Molecules* **2017**, *22*, 1072–9.

[53] I. Vallet-Gély, A. Novikov, L. Augusto, P. Liehl, G. Bolbach, M. Péchy-Tarr, P. Cosson, C. Keel, M. Caroff, B. Lemaitre, *Appl Environ Microbiol* **2010**, *76*, 910–921.

[54] M. Fischer-Le Saux, V. Viallard, B. Brunel, P. Normand, N. E. Boemare, *Int. J. Syst. Bacteriol.* **1999**, *49 Pt 4*, 1645–1656.

[55] N. J. Tobias, B. Mishra, D. K. Gupta, R. Sharma, M. Thines, T. P. Stinear, H. B. Bode, *BMC Genomics* **2016**, *17*, 537.

[56] P. Tailliez, S. Pages, N. Ginibre, N. Boemare, *Int J Syst Evol Microbiol* **2006**, *56*, 2805–2818.

[57] E. I. Vivas, H. Goodrich-Blair, *J. Bacteriol.* **2001**, *183*, 4687–4693.

[58] M. Gualtieri, J.-C. Ogier, S. Pages, A. Givaudan, S. Gaudriault, *Genome Announcements* **2014**, *2*, DOI 10.1128/genomeA.00190-14.

[59] N. Vodovar, D. Vallenet, S. Cruveiller, Z. Rouy, V. Barbe, C. Acosta, L. Cattolico, C. Jubin, A. Lajus, B. Segurens, et al., *Nat Biotechnol* **2006**, *24*, 673–679.

Strain	Oligonucleotide	Sequence (5´- 3´)	Plasmid in *E. coli* S17-1λpir	construction method	NP	Reference
***Photorhabdus laumondii* TT01**						
TT01Δ*hfq*						Tobias et al, 2017
TT01 Δhfq::P_{BAD}-plu2670					kolossin	(Bode et al 2015b)
TT01 Δ*hfq*::P_{BAD}-plu3263					GameXPeptide	Bode et al., 2012; Nollmann et al.
TT01 Δ*hfq*::P_{BAD}-*plu1881*	CEP_Gli_Fw_NdeI	CATATGATGGGCTGGAATATATTTATTAAC	pCEP_kan_*plu1881*	5	glidobactin	this study
	CEP_Gli_Rv_SacI	GAGCTCGTCACGATGAAAACAGTTAGGC				
	V_CEP_Gli_Rv	CTATCTAACGTACCACGCAAACG				
TT01 Δ*hfq*::P_{BAD}-*plu3123*	CEP_plu3123_NdeI	CATATGAAAGATAGCATCGCTCAAGCAG	pCEP_kan_*plu3123*	5	ririwpeptide	this study
	CEP_plu3123_SacI	GAGCTCATCACACGGTCTATCCTCCG				
	V_CEPplu3123	CATAGGTGTCATTAAAACCCAGATAAC				
***Photorhabdus* PB45.5**						
PB45.5 Δ*hfq*	AHp312	GATCCTCTAGAGTCGACCTGCAGAGCTTTATCTATCGCTTTACG	pDelta_PB455_*hfq*			this study
	AHp313	CCGCTCTCAAGCTCTATATTTTCCTTATTTTGTTGTTTTTAAC				
	AHp314	GGAAAATATAGAGCTTGAGAGCGGTAATAGAATG				
	AHp315	AATTCCCGGGAGAGCTCAGATCTATATGCAGCAACAATGTTGC				
	AHp316	CCTTCTATTCGTTGTGTTGG				
	AHp317	ATTTGCACAGGAACTACACC				
	AHp318	AACGGCAATTTTTGTTATGG				
	AHp319	CCAGTACGCTGTCAACAGC				
PB45.5 Δ*hfq*::P_{BAD}-*ct83_145-147*	FF_261	TTTTTTGGGCTAACAGGAGGCTAGCATATGAAAGATAGCATTACTAAAAAAG	pCEP_kan_*ct83_145-147*	2	kolossin	this study
	FF_262	GAGCTCGATATCGCATGCGGTACCTCTAGAACAGATACCCCCCACTATC				
	FF_281	GCTGTGCACCTTCTTTAACACC				
PB45.5 Δ*hfq*::P_{BAD}-*ct87_6*	FF_263	TTTTTTGGGCTAACAGGAGGCTAGCATATGAAAGATAGCGTTGTTAAACAAG	pCEP_kan_*ct87_6*	2	silathride	this study
	FF_264	GAGCTCGATATCGCATGCGGTACCTCTAGATCAGACGATTTACATCATAGATCC				
	FF_282	GGGCTATGACATCCAATGTCTG				
PB45.5 Δ*hfq*::P_{BAD}-*ct87_7*	FF_265	TTTTTTGGGCTAACAGGAGGCTAGCATATGAAAGATAGCATTGTTCAAG	pCEP_kan_*ct87_7*	2	ririwpeptide	this study
	FF_266	GAGCTCGATATCGCATGCGGTACCTCTAGACTTCTGTACTTAGGCGATTTTC				
	FF_283	GGAATGTCTTATTCCGGAGCA				
PB45.5 Δ*hfq*::P_{BAD}-*ct87_8-10*	FF_267	TTTTTTGGGCTAACAGGAGGCTAGCATATGAAAGGTAACATTGCTACAGC	pCEP_kan_ct87_8-10	2	flesuside	this study
	FF_268	GAGCTCGATATCGCATGCGGTACCTCTAGAACAAATATCCCCCCCAATC				
	FF_284	GCGGTATTGGATGTAATAATGTCC				
X. doucetiae						
DSM17909Δ*hfq*	DA_01_rv	TCCCGGGAGAGCTCAGATCTCGTCATCCACCTCTATACG	pEB17_DSM17909Δ*hfq*			this study
	DA_02_fw	CTAAGGGGCAATCTCTGCAAGATCCAGCACAACAGGAAAGCGAT				
	DA_03_fw	CCTCTAGAGTCGACCTGCAGGTCTTCCGGTTGAATTAATC				
	DA_04_rv	CAGCGCCATCGCTTTCCTGTTGTGCTGGATCTTGCAGAGATTGC				
	VP_DA_05-fw	GTGGGTAGTGAGCATGAAAC				
	VP_DA_06-rv	CTTCATCACGATCAATACGG				
	VPEB_289-fw	CCTCCATTCGTTGTGTTG				
	VPEB_290-rv	CCAGAACGGCTAACTCTCC				
DSM17909_Δ*hfq*_P_{BAD}_*gxpS*_km	pCEP_Xdv3_110151_fw	TTTGGGCTAACAGGAGGCTAGCAT_ATGAAAGATAGCAGGGCTAA	pCEP_gxpS_KM	4	GameXPeptide	this study
	pCEP_Xdv3_110151_rv	TCTGCAGAGCTCGAGCATGCACAT_TGGTTTGTGTATCGGTGAG				
	V_Xdv3_110151	CGGAGGTGTACATGATGTAC				
DSM17909_Δ*hfq*_P_{BAD}_*xrdA*_km	pCEP_xr1_pt_fw	TTTGGGCTAACAGGAGGCTAGCATATGCAGCTTTTTCTCAAAG	pCEP_xrdA_KM	4	xenorhabdin	this study
	pCEP_xr1_pt_rv	TCTGCAGAGCTCGAGCATGCACATATGAATATCGACAAACATCAAG				
	V_Xdv3_10499	GGTGGAAACTGCTGTTATCAGGT				
DSM17909_Δ*hfq*_P_{BAD}_*xabA*_km	pCEP_Xdv3_70458_fw	TTTGGGCTAACAGGAGGCTAGCATATGCCTATGTCATGCAATGGTA	pCEP_xabA_KM	4	xenoamicin	this study
	pCEP_Xdv3_70458_rv	TCTGCAGAGCTCGAGCATGCACATATGATGACAACAGAACTGCCAG				
	V_Xdv3_70458	TGTGAGTGTAAATAGGCATG				
DSM17909_Δ*hfq*_P_{BAD}_*DC*_km	pCEP_Xdv3_70305_fw	TTTGGGCTAACAGGAGGCTAGCAT_ATGACTAAACCGTTTCATCC	pCEP_DC_KM	4	acylamide	paper
	pCEP_Xdv3_70305_rv	TCTGCAGAGCTCGAGCATGCACAT_TGGATTGTGGAACCAATAAC				
	V_Xdv3_70305	TGCTCATTCAGGAGCATGG				
DSM17909_Δ*hfq*_P_{BAD}_*xcn*A_km	pCEP_XDD1_RS08255_fw	TTTGGGCTAACAGGAGGCTAGCATATGAAGAAGACGATTTTTGAGTTG	pCEP_xcnA_KM	4	xenocoumacin	this study
	pCEP_XDD1_RS08255_rv	TCTGCAGAGCTCGAGCATGCACATCCGTTAATCGTGCTGTCAC				
	VP_pCEP_XDD1_RS08255	CTCCAACGGTTGAGCATAG				
DSM17909_Δ*hfq*_P_{BAD}_PAX_km	XDV3_70854_fw_gib	TTTGGGCTAACAGGAGGCTAGCAT_ATGAATTACCCGGAAGAATTG	pCEP_pax_KM	4	PAX	this study
	XDV3_70854_rv_gib	TCTGCAGAGCTCGAGCATGCACAT_GTTTTGCCAGAATTGTTGCT				
	V_xdv3_70854_rv	CTGGCGAACGGGTACTTCTG				
X. szentirmaii						
X. szentirmaii Δhfq	ΔXSZ_hfq_up_PstI-Gib_fw	CCTCTAGAGTCGACCTGCAGGGGAGAATTCCACTCCTGG	pCK_cipB_Δ*hfq*_XSZ (pNN06)			
	ΔXSZ_hfq_up_Gib_rev	GGTTCGCACCTAAGGCGTTCAGGAATGG				
	ΔXSZ_hfq_do_Gib_fw	GAACGCCTTAGGTGCGAACCCAGCTGTAC				
	ΔXSZ_hfq_do_BglII-Gib_rev	TCCCGGGAGAGCTCAGATCTGGGTAAATGACGGATGAAACC				
	delta_hfq__XSZ_mut_ver_fw2	GACAGCCATTTTCATTATGG				
	delta_hfq__XSZ_mut_ver_rev2	TCATTACCAACATGGATGG				
X. szentirmaii Δ*hfq* pCEP-KM-1979	MHp274	GGCTAACAGGAGGCTAGCATATGAAAGTAAGAAAAGATATTGCTG	pCEP-KM-1979	1	szentirazin	this study
	MHp275	GCCGTTTAAACATTTAAATCTGCAGCTAATTCCTGATTCATCGAC				
	MHp273	CTTCTACACCTGAGGCAGATG				
X. szentirmaii Δ*hfq* pCEP-KM-3460	MHp279	GGCTAACAGGAGGCTAGCATATGAAAGGTAGTATTGCTAAAAAGG	pCEP-KM-3460	1	szentiamid	this study
	MHp280	GCCGTTTAAACATTTAAATCTGCAGGGTGAGTTACCAATGTGTCC				
	MHp281	CCAGCATCAGGACGATGC				
X. szentirmaii Δ*hfq* pCEP-KM-3680	MHp285	GGCTAACAGGAGGCTAGCATATGATAAAATCTGAATACATAATAA	pCEP-KM-3680	1	xenobactin	this study
	MHp286	GCCGTTTAAACATTTAAATCTGCAGGGTGACAGAATCTAAATATTGT				
	MHp287	ATTGGTGTGGCTCATGTTC				
X. szentirmaii Δ*hfq* pCEP-KM-0377	MHp294	GGCTAACAGGAGGCTAGCATATGAATTACCCTGAAAAGTTGA	pCEP-KM-0377	1	PAX-short	this study
	MHp295	GCCGTTTAAACATTTAAATCTGCAGCATTGTGATACGGCTAAATC				
	MHp296	GCCATCGCTTGCAGTTTC				
X. szentirmaii Δ*hfq* pCEP-KM-3942	MHp297	GGCTAACAGGAGGCTAGCATATGGAACATTTTGACATAGATAA	pCEP-KM-3942	1	rhabduscin	this study
	MHp298	GCCGTTTAAACATTTAAATCTGCAGGATACAATCTGACTCCCTCTT				
	MHp299	CAGCTACGCTGGATGACAC				
X. szentirmaii Δ*hfq* pCEP-KM-3397	MHp306	GGCTAACAGGAGGCTAGCATATGAAAAATGCTGCTCAAATTG	pCEP-KM-3397	1	rhabdopeptide	this study
	MHp307	GCCGTTTAAACATTTAAATCTGCAGTGTGACCAGCTATCACAAGC				
	MHp308	CGCTGCCATTGTGCATAAT				
X. szentirmaii Δ*hfq* pCEP-KM-0346	X.sz_Cl2_ORF00346_fw_gib_LP	TTTGGGCTAACAGGAGGCTAGCATATGAAAGATAGCAAGGTTGCT	pCEP-KM-0346	3	GameXPeptide	this study
	X.sz_Cl2_ORF00346_rv_gib_LP	TCTGCAGAGCTCGAGCATGCACATGGTATACATGATGTACGCTGG				
	X.sz_Cl2_ORF00346_ver_rv_LP	GATAACGAGCCGGACTACAGC				
X. szentirmaii Δ*hfq* pCEP-KM-3636	X.sz_Cl11_1st_ORF03636_fw_gib_LP	TTTGGGCTAACAGGAGGCTAGCATATGTCTATGTCATGTCACCGT	pCEP-KM-3636	3	xenoamicin	this study
	X.sz_Cl11_ORF03636_rv_gib_LP	TCTGCAGAGCTCGAGCATGCACATCCAGAACTGCTTGTCTTGTATA				
	X.sz_Cl11_ORF03636_ver_rv_LP	GCTGGCTGACTTCAGTGGATAG				
X. szentirmaii Δ*hfq* pCEP-KM-5118	X.sz_Cl16_ORF05118_fw_gib_LP	TTTGGGCTAACAGGAGGCTAGCATATGTACACATTACAGGCATTACC	pCEP-KM-5118	3	pyrollizizenamid	this study
	X.sz_Cl16_ORF05118_rv_gib_LP	TCTGCAGAGCTCGAGCATGCACATGCTGTGACAGAATTTCTTCTT				
	X.sz_Cl16_ORF05118_ver_rv_LP	CCTGAGTTAATATATGCCGGTGT				
X. szentirmaii Δ*hfq* pCEP-KM-*fclC*	X.sz_Cl21_ORF06379_fw_gib_LP	TTTGGGCTAACAGGAGGCTAGCATATGGTGATTTCAACTGAATTC	pCEP-KM-*fclC*	3	fabclavin	this study
	X.sz_Cl21_ORF06379_rv_gib_LP	TCTGCAGAGCTCGAGCATGCACATCGTAGTATCTTCTGCTGTCAG				
	X.sz_Cl21_ORF06379_ver_rv_LP	GAGCTGACTGACTTTATCCCAC				
***X. nematophila* HGB081**						
X. nematophila Δ*hfq*	NNp13	CCTCTAGAGTCGACCTGCAGCGCAGAAGAACAAGCTCAGG	pCKcipB_Delta*XNC1_0455*			
	NNp14	GTGATAGTTACCGACCCTTTCACGCCGTAAAG				
	AHp158	CGTGAAAGGGTCGGTAACTATCACGCGGGTTC				
	AHp159	TCCCGGGAGAGCTCAGATCTCTGTTTCTCAACTTTGCTGAGG				
	delta_hfq__XNC1_mut_ver_fw2	TGAAGTGAAAGCACTTTATGC				
	delta_hfq__XNC1_mut_ver_rev2	AATATTTTGGATGAGGTGCC				
X. nematophila Δ*hfq* PAX	DAS_35_ fw	TTTGGGCTAACAGGAGGCTAGCATATGAATTACCCTGAAACCT	pCEP_kan_*XNC1_2783*	4	PAX	this study
	DAS_36_ rv	TCTGCAGAGCTCGAGCATGCACATGGCTGTTATCATACTTAAGC				
	DA _37_VP_ rv	CTCTCCGACCAGTTTTCCC				
X. nematophila Δ*hfq* pCEP-KM-Xenoamicin	DAS_29_ fw	TTTGGGCTAACAGGAGGCTAGCATATGTCTATGTCATGCAATC	pCEP_kan_*XNC1_2040*	4	xenoamicin	this study
	DAS_30_ rv	TCTGCAGAGCTCGAGCATGCACATCAGCCACAGTCTCTTCTAT				
	DA _31_ VP_ rv	GCCGATGACAATCTCTTCTGC				
X. nematophila Δ*hfq* pCEP-KM-Xenocoumacine	DAS_26_ fw	TTTGGGCTAACAGGAGGCTAGCATATGAAGAAGACGATTTTTG	pCEP_kan_*XNC1_1711*	4	xenocoumacin	this study
	DAS_27_ rv	TCTGCAGAGCTCGAGCATGCACATTAATGTACACGCTTGGATC				
	DA _28_VP_rv	CTATACGCAAATGGTCATTGAAGATG				
X. nematophila Δ*hfq* pCEP-KM-Xenortid	DAS_32_ fw	TTTGGGCTAACAGGAGGCTAGCATATGAAAAATGCTGCGCAAA	pCEP_kan_*XNC1_2300*	4	xenortide	this study
	DAS_33_ rv	TCTGCAGAGCTCGAGCATGCACATCTGAAATCCACGTGAAAGATA				
	DA _34_VP_fw	CTTGAATGAATGGCTCTTCTGC				
X. nematophila Δ*hfq* pCEP-KM-Xenotetrapeptide	DAS_23_ fw	TTTGGGCTAACAGGAGGCTAGCATATGAAAGATAGCATGGCTA	pCEP_kan_*XNC1_2022*	4	xenotetrapeptide	this study
	DAS_24_ rv	TCTGCAGAGCTCGAGCATGCACATGGTCATTGGTTTCAATTTC				
	DA - 25- VP- rv	TGCCTGTACAAATTCCGGG				
***Pseudomonas entomophila* L48**						
*P. entomophila*Δ*hfq*	PEB_534-fw	CCTCTAGAGTCGACCTGCAGCCAAGGATCCTGATTGTTCG	pEB17_Pseen_Δ*hfq*			this study
	PEB_535-rv	GTTTTTCTACTATTGATCCTTACAGGAGTGGAGCCTGCATTGTTCTTTGAGC				
	PEB_536-fw	CGTGGCGCTCAAAGAACAATGCAGGCTCCACTCCTGTAAGGATCAATAGTAG				
	PEB_537-rv	TCCCGGGAGAGCTCAGATCTGAGCTGATCAGCGTCGATTC				
	VPEB_538-fw	GAACATGCCGTTCAGGCTGTC				
	VPEB_539-rv	GAGTCCCTGCTTATAGCCGTC				
*P. entomophila*Δ*hfq*_P_{BAD}#5	PEB_554-fw	TTTGGGCTAACAGGAGGCTAGCATATGTTCCAGATATACAGCTGC	pCEP_Pseen_XDD1_RS10885_km	4	pyreudiones	this study
	PEB_555-rv	TCTGCAGAGCTCGAGCATGCACATGTCTCACCCTGGAACACCTG				
	VPEB_556-rv	GGTCATGCGGTGCAGGTAG				
*P. entomophila*Δ*hfq*_P_{BAD}#7	PEB_518-fw	TTTGGGCTAACAGGAGGCTAGCATATGCCCGACACTTCCTCCTTG	pCEP_Pseen_XDD1_RS12600_km	4	pseudotetrapeptide	this study
	PEB_519-rv	TCTGCAGAGCTCGAGCATGCACATCTGCAACGGGTACAGCCGTGC				
	VPEB_520-rv	GCTGAACACATCGAACGAG				
*P. entomophila*Δ*hfq*_P_{BAD}_etlA	PEB_557-fw	TTTGGGCTAACAGGAGGCTAGCAT_ATGTTCGCCGCCGACACATAC	pCEP_Pseen_XDD1_RS15405_km	4	entolysin short	this study
	PEB_558-rv	TCTGCAGAGCTCGAGCATGCACAT_CAGCAGCACATCCGGCAAC				
	VPEB_570-rv	CCAAAGTCCATGCGCAAC				
